# LINCs Are Vulnerable to Epileptic Insult and Fail to Provide Seizure Control via On-Demand Activation

**DOI:** 10.1523/ENEURO.0195-22.2022

**Published:** 2023-02-14

**Authors:** Bethany J. Stieve, Madison M. Smith, Esther Krook-Magnuson

**Affiliations:** 1 Graduate Program in Neuroscience, University of Minnesota, Minneapolis, Minnesota 55455; 2 Department of Neuroscience, University of Minnesota, Minneapolis, Minnesota 55455

**Keywords:** channelrhodopsin, closed-loop, GABAergic, interneuron, nNOS, responsive neurostimulation

## Abstract

Temporal lobe epilepsy (TLE) is notoriously pharmacoresistant, and identifying novel therapeutic targets for controlling seizures is crucial. Long-range inhibitory neuronal nitric oxide synthase-expressing cells (LINCs), a population of hippocampal neurons, were recently identified as a unique source of widespread inhibition in CA1, able to elicit both GABA_A_-mediated and GABA_B_-mediated postsynaptic inhibition. We therefore hypothesized that LINCs could be an effective target for seizure control. LINCs were optogenetically activated for on-demand seizure intervention in the intrahippocampal kainate (KA) mouse model of chronic TLE. Unexpectedly, LINC activation at 1 month post-KA did not substantially reduce seizure duration in either male or female mice. We tested two different sets of stimulation parameters, both previously found to be effective with on-demand optogenetic approaches, but neither was successful. Quantification of LINCs following intervention revealed a substantial reduction of LINC numbers compared with saline-injected controls. We also observed a decreased number of LINCs when the site of initial insult (i.e., KA injection) was moved to the amygdala [basolateral amygdala (BLA)-KA], and correspondingly, no effect of light delivery on BLA-KA seizures. This indicates that LINCs may be a vulnerable population in TLE, regardless of the site of initial insult. To determine whether long-term circuitry changes could influence outcomes, we continued testing once a month for up to 6 months post-KA. However, at no time point did LINC activation provide meaningful seizure suppression. Altogether, our results suggest that LINCs are not a promising target for seizure inhibition in TLE.

## Visual Abstract

**Figure:**
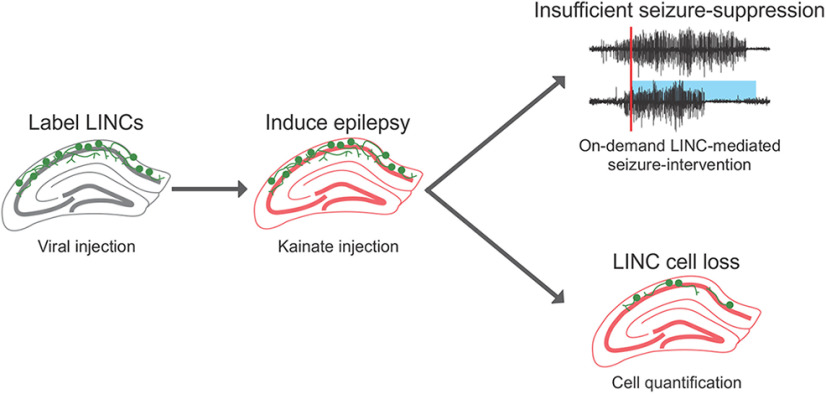

## Significance Statement

Novel treatments are needed for temporal lobe epilepsy, and altering inhibitory signaling may provide seizure control. Recently, a previously uncharacterized hippocampal cell population, long-range inhibitory neuronal nitric oxide synthase-expressing cells (LINCs), was found to provide strong, widespread inhibition in healthy tissue. Despite being a novel source of powerful inhibition, and therefore a promising candidate for seizure control, on-demand activation of LINCs in a mouse model of temporal lobe epilepsy did not substantially suppress seizure activity. LINC numbers were decreased in epileptic tissue, indicating that LINCs are a vulnerable cell population. The heterogeneity of inhibitory signaling, and how it changes in epilepsy, are important factors to consider when developing new temporal lobe epilepsy therapies.

## Introduction

Temporal lobe epilepsy (TLE), characterized by spontaneous recurrent seizures typically arising from the hippocampus, is the most common form of epilepsy in adults (England et al., 2012). With current antiseizure drugs, only 11–25% of TLE patients are seizure free (Zumsteg, 2006; Löscher and Schmidt, 2011; Bialer et al., 2020). Additionally, those who can find some relief may experience a range of negative side effects including nausea, liver dysfunction, bone degradation, and chronic cognitive impairments (Spuck et al., 2010; de Tisi et al., 2011; Sherman et al., 2011; Perucca and Gilliam, 2012; Duchowny and Bhatia, 2014; Laxer et al., 2014). Dysregulation of GABAergic signaling is a known key ictogenic factor (Hawkins and Sarett, 1957; Benassi, 1962; Krnjević, 1983). However, the limited efficacy and negative side effects of antiseizure drugs that indiscriminately elevate inhibitory signaling emphasize the need to consider the timing of interventions, circuit changes in epilepsy, and the heterogeneity of GABAergic signaling to develop high-specificity treatments that can provide seizure control while limiting side effects (Muldoon et al., 2015; Christenson Wick and Krook-Magnuson, 2018; Farrell et al., 2019; Moxon et al., 2019; Dudok et al., 2022). There is incredible diversity of GABAergic signaling and GABAergic neurons (DeFelipe et al., 2013; Lovett-Barron and Losonczy, 2014; Pelkey et al., 2017; Wamsley and Fishell, 2017; Muñoz-Manchado et al., 2018). For example, in CA1, a subregion of the hippocampus, there are >20 types of inhibitory neurons identified (Freund and Buzsáki, 1996; Klausberger and Somogyi, 2008). Recent work has identified and characterized additional CA1 populations, and genomic analysis suggests that even more populations remain poorly characterized (Francavilla et al., 2018; Harris et al., 2018; Christenson Wick et al., 2019; Yao et al., 2021; Szabo et al., 2022). Each interneuron subtype has distinct properties and roles, subsequently forming unique microcircuits in healthy and epileptic tissue (Alexander et al., 2016; Booker and Vida, 2018; Shuman et al., 2020; Dudok et al., 2021; Righes Marafiga et al., 2021).

This heterogeneity in inhibitory cell populations provides both a challenge and an opportunity. It may be possible to harness the diversity of interneurons to identify novel and effective targets for seizure intervention. On-demand optogenetic seizure intervention (Armstrong et al., 2013; Paz et al., 2013) enables cell type-specific manipulations (Krook-Magnuson and Soltesz, 2015) and is a useful approach to study the potential for specific cell populations to control seizures (for review, see Tønnesen and Kokaia, 2017; Christenson Wick and Krook-Magnuson, 2018). This approach not only limits manipulation to specific cell populations, but also limits the timing of intervention to only when a seizure is detected (i.e., closed-loop stimulation, also known as responsive stimulation; Nagaraj et al., 2015).

Optogenetic approaches have been used to explore hippocampal circuit elements, and specifically, which populations may be able to curtail (or worsen or even induce) temporal lobe seizures (Krook-Magnuson et al., 2013; Osawa et al., 2013; Sukhotinsky et al., 2013; Berglind et al., 2014; Chiang et al., 2014; Krook-Magnuson et al., 2015; Ladas et al., 2015; Weitz et al., 2015; Wang et al., 2017; Bui et al., 2018; Chen et al., 2018, 2021; Tung et al., 2018; Botterill et al., 2019; Streng and Krook-Magnuson, 2020; Streng et al., 2021). This includes examination of inhibitory cell populations, such as parvalbumin-expressing cells (PV cells). Hippocampal PV cells encompass several inhibitory cell types and subtypes, including basket, axoaxonic, and bistratified cells (Woodson et al., 1989; Freund and Buzsáki, 1996; Varga et al., 2014). While the role of PV cells in seizures appears to be situation specific (Ellender et al., 2014; Sessolo et al., 2015; Assaf and Schiller, 2016; Lévesque et al., 2019; Magloire et al., 2019; Dudok et al., 2022; Wang et al., 2022), and may not be the same for all subtypes (Christenson Wick and Krook-Magnuson, 2019), previous work using on-demand optogenetic activation of PV cells at the onset of seizures in the intrahippocampal kainate (IHKA) mouse model of chronic TLE demonstrated successful hippocampal seizure inhibition (Krook-Magnuson et al., 2013, 2014b; Chen et al., 2021). This previous work supports the idea that *in vivo* activation of a select subpopulation of interneurons can be sufficient for the suppression of hippocampal seizures. However, the degree of seizure inhibition mediated by activation of PV cells was not as high as observed with some other closed-loop cell manipulations in the same TLE model (Krook-Magnuson et al., 2013, 2014b; Streng and Krook-Magnuson, 2020; Streng et al., 2021). Targeting different populations of hippocampal interneurons might be able to outperform on-demand optogenetic activation of hippocampal PV cells.

Recently, a previously uncharacterized source of widespread inhibition in the hippocampus was described—long-range inhibitory neuronal nitric oxide synthase (nNOS)-expressing cells (LINCs; Christenson Wick et al., 2019). LINCs are a GABAergic neuronal population in CA1, and, because of the following several unique features, we hypothesized that LINCs would be in a privileged position to provide seizure control. (1) Despite being a relatively sparse inhibitory cell population, LINCs provide widespread inhibition within CA1. Optogenetic activation of LINCs in hippocampal slices resulted in postsynaptic inhibition of 80% of all patched cells in CA1, including both superficial and deep pyramidal cells (Christenson Wick et al., 2019). For comparison, PV cells primarily target deep pyramidal cells (Lee et al., 2014). (2) LINCs are capable of altering hippocampal oscillations *in vivo* and therefore might also be effective in controlling aberrant network seizure activity (Christenson Wick et al., 2019). (3) LINCs produce both GABA_A_ and GABA_B_ postsynaptic responses (Christenson Wick et al., 2019). Compared with GABA_A_, GABA_B_-mediated inhibition is not only longer lasting, but also independent of the intracellular chloride concentration (which may be altered in epilepsy, reducing the efficacy of GABA_A_-mediated inhibition; Cossart et al., 2005; Miles, 2012; Alfonsa et al., 2015; Wang et al., 2017).

Despite the widespread inhibition provided by LINCs in healthy CA1, they have never been studied in the context of epilepsy (as they have only recently been described in healthy animals). Recent investigation of LINCs (in healthy animals) was made possible by advances in viral vectors using intersectional genetic approaches (Fenno et al., 2014). LINCs are identified in part by their expression of nNOS (Christenson Wick et al., 2019). However, nNOS is also expressed by excitatory neurons (Burette et al., 2002), which far outnumber LINCs. Thus, it was only with a dual-targeting strategy that the investigation of LINCs could be achieved: specifically, as done previously (Christenson Wick et al., 2019), we targeted LINCs via their expression of both nNOS and Dlx5/6 (a transcription factor expressed by GABAergic neurons of the forebrain; Dimidschstein et al., 2016). While other interneurons can also express nNOS (Jinno et al., 2007; Tricoire et al., 2010; Krook-Magnuson et al., 2011; Armstrong et al., 2012), the viral vector approach taken, coupled with the location of injection, preferentially captures hippocampal LINCs (Christenson Wick et al., 2019).

To determine whether LINCs can influence seizure activity, we used the IHKA mouse model of chronic TLE (Bouilleret et al., 1999; Zeidler et al., 2018) and optogenetically activated LINCs *in vivo* for on-demand seizure intervention. LINC-mediated seizure intervention was tested once a month, starting at 1 month post-epilepsy induction, for up to 6 months. We found that LINC activation did not substantially change seizure duration, even at late time points. We additionally found a substantial reduction in the number of LINCs in epileptic tissue, suggesting epilepsy-related cell death. A reduction in the number of hippocampal LINCs was observed even when the site of KA injection was moved to the amygdala. A propensity of LINC cell death in temporal lobe epilepsy may prevent meaningful seizure inhibition via on-demand optogenetic activation of LINCs and warrants future work examining the impact of this cell loss on hippocampal function.

## Materials and Methods

### Animals

All animal procedures were performed in accordance with the University of Minnesota animal care and use committee regulations. At all times, mice had *ab libitum* access to food and water and were on a 12 h light/dark cycle (/low red light; zeitgeber time 0 = 8:00 A.M.). Both male and female mice, sexed at the time of weaning based on external genitalia, were used in all experiments. Mice were group housed in standard housing conditions in the animal facility at the University of Minnesota before and after viral injections. When needed, male mice were singly housed to avoid fighting after KA injections. Following implant surgery, all mice were singly housed to avoid damage to implants. Mice lived in investigator-managed housing during electrographic recordings.

**Figure 1.**
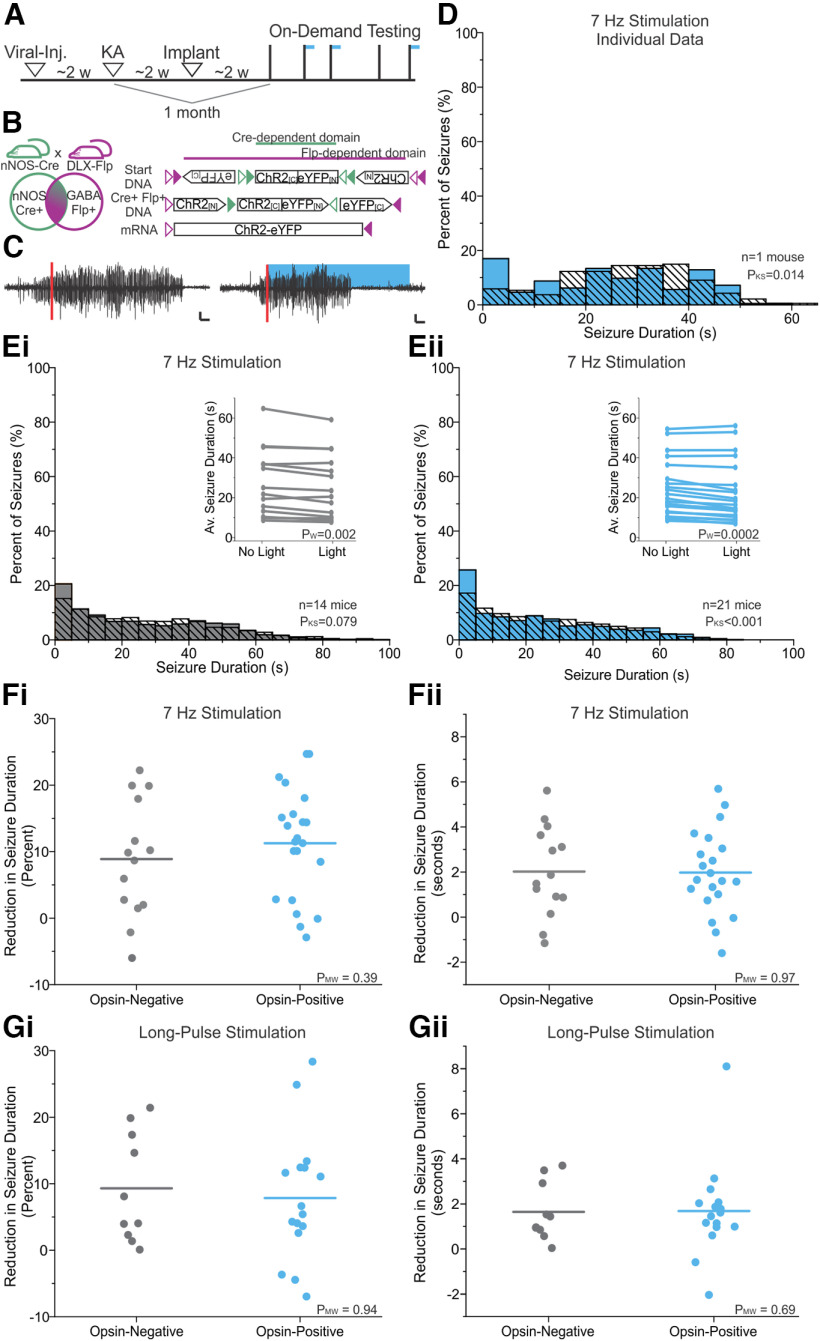
One month post-epilepsy induction, on-demand activation of LINCs does not provide strong seizure control. ***A***, Experimental timeline. On-demand seizure intervention starts at least 6 weeks post-viral injection and 1 month post-KA injection to induce epilepsy. During on-demand testing, the hippocampal LFP is continuously monitored and analyzed online for seizure detection (indicated by vertical lines), which triggers pulsed blue light delivery for 50% of seizures in a random manner (horizontal blue lines). ***B***, An intersectional genetic viral vector approach is used to label LINCs, an nNOS-expressing GABAergic cell population. Double-expressing offspring of nNOS-Cre and Dlx5/6-Flpe mice (left) are injected with a Cre-dependent and Flp-dependent virus (right) to mediate expression of eYFP-tagged channelrhodopsin for optogenetic activation and fluorescent labeling of LINCs. Flp-negative littermates receive a viral injection in the same manner and are used as opsin-negative controls. Schematics were adapted from the studies by Christenson Wick et al. (2019) and Fenno et al. (2014). ***C***, Example electrographic seizures detected (red vertical line) from an opsin-positive mouse, triggering on-demand seizure intervention (right trace; 7 Hz blue light stimulus for 30 s) or not receiving light (left trace; used as an internal control). Scale: 500 μV, 2 s. ***D***, Post-detection seizure durations during light (solid blue) and no-light (hashed black) conditions from an animal with a statistically significant—but still very minor—effect of light (seizure duration reduction: 12%, 2.5 s; *p* < 0.05, MW and KS tests). ***Ei***, ***Eii***, Postdetection seizure duration distributions at the group level. Solid gray bars (control animals; ***Ei***) or blue bars (opsin-positive animals; ***Eii***), seizures receiving 7 Hz light stimulation; black hashed bars, no-light seizures. Insets, Average seizure duration during no-light and light conditions by animal. ***Fi***, ***Fii***, Reduction in seizure duration by animal, as percentage of seizure duration reduction (***Fi***) or difference in seizure duration in seconds (***Fii***) for light (7 Hz stimulation) versus no light. Gray dots, control animals; blue dots, opsin-positive animals. ***G***, Same as ***F***, but for long-pulse light delivery (1 s on, 50 ms off, for 3 s). Note that while some individual animals showed a statistically significant response to light (***D***, example), and while there is a slight decrease in the overall seizure duration distribution in opsin-positive animals (***Eii***), there is no difference between opsin-positive and opsin-negative animals in effect size across animals (***F***, ***G***), and no animal shows a strong response to light (maximum percentage seizure reduction is <30%; Table 1, summary of statistical analyses).

For all experiments, mice were bred in-house. Using an intersectional genetic approach (Fenno et al., 2014), we virally labeled the nNOS-expressing GABAergic cell population LINCs, as described in the study by Christenson Wick et al. (2019; Fig. 1*B*, Table 1). To enable this approach, two lines were crossed. One line expressed Cre-recombinase in nNOS-expressing neurons and is referred to here simply as “cNOS”. The second line expressed Flpe recombinase in GABAgeric forebrain neurons via the *Dlx5/Dlx6* (*id6/id5*) intergenic enhancer region and beta-globin basal promoter, and here is referred to simply as “fDlx.” Crossing these lines resulted in “cNOS-fDLX” mice, which included offspring expressing both Cre and Flp recombinases in nNOS-expressing GABAergic neurons of the forebrain. Double-expressing cNOS-fDLX mice (Cre-positive and Flp-positive) were injected with virus and used as opsin-positive animals. Flp-negative cNOS-fDLX littermates underwent identical surgical procedures including viral injections. As viral expression requires both Cre and Flp expression, Flp-negative animals did not express channelrhodopsin and were used as opsin-negative controls. nNOS mice were purchased from The Jackson Laboratory (B6.129-*Nos^1^*^tm1(cre)Mgmj^/J; stock #017526; Leshan et al., 2012) and were maintained as homozygotes. Founder fDLX mice were provided by the Fishell Laboratory, Harvard Medical School, Cambridge, MA, and are also available from The Jackson Laboratory [Tg(ml56i-flpe)39Fsh/J, stock #010815; Miyoshi et al., 2010)]. fDLX breeders were backcrossed at least eight times with C57BL/6J mice (stock #000664; The Jackson Laboratory; bred in-house), and were maintained as hemizygotes. C57BL/6J mice were used for PV cell quantification (see below).

**Table 1:** Statistical analyses

Reference	Test	*n*	*p*-value
Fig. 1*D*	Kolmogorov–Smirnov	194 light, 187 no-light seizures	0.014
Fig. 1*Ei*, inset	Paired Wilcoxon	14 mice	0.002
Fig. 1*Ei*	Kolmogorov–Smirnov	14 mice	0.079
Fig. 1*Eii*, inset	Paired Wilcoxon	21 mice	0.0002
Fig. 1*Eii*	Kolmogorov–Smirnov	21 mice	3.6889E-8
Fig. 1*Fi*	Mann–Whitney	14 opsin-negative, 21 opsin-positive mice	0.39
Fig. 1*Fii*	Mann–Whitney	14 opsin-negative, 21 opsin-positive mice	0.97
Fig. 1*Gi*	Mann–Whitney	10 opsin-negative, 16 opsin-positive mice	0.94
Fig. 1*Gii*	Mann–Whitney	10 opsin-negative, 16 opsin-positive mice	0.69
Fig. 1*Fi–Gii*	Two-way ANOVA	14 opsin-neg., 7 Hz stim; 21 opsin-pos., 7 Hz stim; 10 opsin-neg., long-pulse stim; 16 opsin-pos., long pulse stim mice	0.66; 0.66; 0.53
Results	Two-way ANOVA	6 opsin-neg., male; 8 opsin-neg., female; 12 opsin-pos., male; 9 opsin-pos., female mice	0.34, 0.07, 0.39
Fig. 2*E*	Kruskal-Wallis ANOVA	7 saline-injected, 6 kainate-injected (intrahippocampal), 5 kainate-injected (intra-amygdala) mice	0.006
Fig. 2*E*	Mann–Whitney	7 saline-injected, 6 kainate-injected (intrahippocampal) mice	0.004
Fig. 2*E*	Mann–Whitney	7 saline-injected, 5 kainate-injected (intra-amygdala) mice	0.015
Results	Mann–Whitney	5 saline-injected, 6 kainate-injected mice	0.008
Results	Mann–Whitney	6 C57BL/6J mice, 10 cNOS-fDLX mice	0.002
Fig. 3*Ai*	Spearman’s correlation	46 on-demand intervention sessions; 21 mice	0.033
Fig. 3*Aii*	Spearman’s correlation	65 on-demand intervention sessions; 21 mice	0.0015
Fig. 3*Bi*	Spearman’s correlation	46 on-demand intervention sessions; 21 mice	0.39
Fig. 3*Bii*	Spearman’s correlation	65 on-demand intervention sessions; 21 mice	0.28
Fig. 3*Ci*	Spearman’s correlation	38 on-demand intervention sessions; 17 mice	0.0063
Fig. 3*Cii*	Spearman’s correlation	51 on-demand intervention sessions; 19 mice	0.00014
Fig. 3*Di*	Spearman’s correlation	38 on-demand intervention sessions; 17 mice	0.056
Fig. 3*Dii*	Spearman’s correlation	51 on-demand intervention sessions; 19 mice	0.013
Materials and Methods	Spearman’s correlation	24 opsin-positive, 20 opsin-negative mice	0.60
Materials and Methods	Spearman’s correlation	7 opsin-positive saline-injected mice	0.38
Results	Spearman’s correlation	46 on-demand intervention sessions; 21 mice	0.12
Results	Spearman’s correlation	65 on-demand intervention sessions; 21 mice	1.95E-5
Results	Spearman’s correlation	38 on-demand intervention sessions; 17 mice	0.078
Results	Spearman’s correlation	51 on-demand intervention sessions; 19 mice	2.57E-5

### Stereotactic surgeries

Mice underwent one to three stereotaxic surgeries conducted under 1–3% isoflurane anesthesia. Unless noted otherwise, mice received treatment with carprofen (5 mg/kg, s.c.), local bupivacaine at the start of surgery, and postsurgical NeoPredef treatment around incisions, and recovered on a heating pad with appropriate observation.

### Viral injections

For all LINC-related experiments, mice first received a viral injection, starting at postnatal day 45 or greater (Fig. 1*A*, full timeline). In cNOS-fDLX-positive mice (described above), LINCs were labeled with the excitatory opsin channelrhodopsin-2 (ChR2) fused to enhanced yellow fluorescent protein (eYFP) in a Cre-dependent and Flp-dependent manner [AAV-DJ-hSyn-Con/Fon-hChR2(H134R)-eYFP-WPRE; titers, 4.4 × 10^12^ and 5.8 × 10^12^; lot #AV6214C and #AV8403, UNC Viral Vector Core; Fig. 1*B*]. One microliter of virus was injected using a microliter syringe (Hamilton 2 μl syringe, Neuros) into CA1 of the left dorsal hippocampus [distance from bregma: anteroposterior (AP), −2.0 mm; mediolateral (ML), 1.25 mm left; dorsoventral (DV), 1.35 mm], at an approximate rate of 200 nl/min. The syringe was left in place for at least 5 min postinjection before being withdrawn. Virus injection with this approach into the stratum oriens preferentially labels LINCs (Christenson Wick et al., 2019); Ivy and neurogliaform cells also express nNOS, but have a spatially restricted axonal plexus (Fuentealba et al., 2008; Armstrong et al., 2012), which likely limits virus uptake. However, any Ivy cells at the site of injection could potentially express eYFP-ChR2 with this approach. Any such off-target labeling should result in a very minimal number of Ivy cells expressing eYFP-ChR2, as they would need to be in close proximity to the injection site. Ivy cells are inhibitory and should, if anything, increase the efficacy of intervention if they inadvertently also expressed ChR2 and received light. Flp-negative littermates were injected in the same manner and with the same virus as cNOS-fDLX-positive mice. All on-demand experiments and tissue collection began at least 6 weeks post-viral injection.

### Epilepsy induction

We primarily used the IHKA mouse model of chronic TLE, in which KA is injected directly into the hippocampus (Bouilleret et al., 1999; Zeidler et al., 2018). In this model, weeks after KA injection, animals display spontaneous, recurrent seizures, which can be recorded electrographically from the hippocampus (Riban et al., 2002). The IHKA epilepsy model replicates key factors of pathology observed in TLE patients, including unilateral hippocampal sclerosis marked by partial cell loss, granule cell dispersion, and mossy fiber spouting (Suzuki et al., 1995; Bouilleret et al., 2000; Twele et al., 2016).

At least 13 d post-viral injection, animals received an intrahippocampal injection of KA. Specifically, 90–100 nl of 18.5 mM KA in sterile saline (SA) was injected with a microliter syringe into the hippocampus (distance from bregma: AP, −2.8 mm; ML, 2.15 mm left; DV, 1.9 mm). Note that this injection site is posterior to the viral injection to avoid direct overlap, and that KA injection was performed at a later time point to allow viral expression before this insult. For PV cell quantification (see below), C57BL/6J mice received an intrahippocampal KA injection in the same manner as cNOS-fDLX mice.

In a separate set of experiments, the intra-amygdala KA model of chronic temporal lobe epilepsy (Ben-Ari et al., 1980; Li et al., 2008; Mouri et al., 2008; West et al., 2022; BLA-KA) was used instead. In these cNOS-fDLX animals, 200 nl of 18.5 mM KA in sterile saline was injected into the basolateral amygdala (distance from bregma: AP, −1.0 mm; ML, 3.25 mm left; DV, 5.0 mm). A subset of BLA-KA mice (three of five mice) underwent electrode and optic fiber implantation (see below). Hippocampal recordings from the three implanted BLA-KA mice all displayed spontaneous recurrent seizures, and one mouse displayed a high enough frequency of seizures to enable on-demand intervention; the majority of seizures detected in this animal during on-demand optogenetic seizure intervention were overtly behavioral.

An additional set of animals was used as controls for eYFP-positive cell counts and PV-immunohistochemistry experiments (see below). For this, opsin-positive cNOS-fDLX or C57BL/6J mice were injected with 90–100 nl of 0.9% sterile saline in the same manner as IHKA injections.

For all epilepsy-induction and saline-control surgeries, mice received local bupivacaine, were rapidly recovered from anesthesia (within 5 min of injection; Twele et al., 2016), were treated with NeoPredef, and received no postsurgical analgesics to avoid interfering with epilepsy induction.

### Electrode and optic fiber implantation

At least 2 weeks post-epilepsy induction, mice were implanted with a recording electrode and an optic fiber. The hippocampal local field potential (LFP) was monitored by a bipolar twisted (local differential) microwire electrode (50 μm diameter stainless steel with polyimide insulation; P1 Technologies) implanted at 2.4 mm posterior, 2.0 mm left, and 1.75 mm ventral to bregma (IHKA mice), or 3.1 mm posterior, 3.6 mm left, and 3.4 mm ventral to bregma (BLA-KA mice). In all mice, an optic fiber, constructed in-house [diameter, 200 μm; 0.39 numerical aperture (NA)], was implanted for light delivery at 2.2 mm posterior, 1.2 mm left, and 1.35 mm ventral to bregma. Mice received standard surgical treatments of carprofen (5 mg/kg, s.c.), local bupivacaine at the start of surgery, and postsurgical NeoPredef treatment around the implant. Additionally, mice were treated with ibuprofen (50–80 mg/kg/d dissolved in drinking water, for 1 presurgical and 3 postsurgical days). Following implant surgery, mice recovered for at least 1 week before the start of electrophysiological recordings.

### Closed-loop seizure detection and optogenetic LINC activation

Simultaneous 24 h video LFP recordings of mice were used for on-demand optogenetic seizure intervention as described in the study by Armstrong et al. (2013). Hippocampal LFP was transmitted via electrical patch cables connected through an electrical commutator (P1 Technologies), amplified by an analog amplifier (model 410, Brownlee), digitized (National Instruments), and analyzed using custom MATLAB software. Online seizure detection was tuned for each animal, based on ictal spiking properties such as spike amplitude, minimum and maximum spike width, and interspike interval. For each seizure detected, there was a 50% chance that blue light (wavelength ∼473 nm; via laser (Shanghai Laser & Optics Century Co., Ltd.) or LED (Plexon)) would be delivered to the hippocampus via an optical patch cord (ThorLabs) through an optical commutator (Doric; Fig. 1*C*, example LFP traces). Thus, for each mouse about half of detected seizures received light, and half did not receive light, in a random manner, providing an internal within-animal comparison. Light was delivered either at 6.67 Hz with a 33.3% duty cycle (50 ms light pulses) for 30 s, or at 1 Hz with a 95% duty cycle (1 s light pulses) for 3 s, matching previous protocols (Krook-Magnuson et al., 2013, 2014b). Previous work supports that LINCs are activated by a train of 50 ms light pulses, which elicits repeated postsynaptic inhibition (Christenson Wick et al., 2019). The power of light delivered through the tip of the implanted fiber, measured *post hoc*, was 4.9 ± 0.4 mW. Across animals, there was no correlation between light power and the percentage of reduction in seizure duration with light (*p* = 0.60, Spearman’s correlation).

On-demand testing in IHKA mice started 1 month post-KA injection. A subset of mice was tested for longer, for which we conducted on-demand testing once a month for up to 6 months post-KA injection. Another subset of mice started at a later time point and were tested monthly up to 6 months post-KA injection. At each time point, mice received two rounds of on-demand testing, one with each light stimulus (7 and 1 Hz stimulation). For each round of intervention, testing was conducted across both light cycles (i.e., across the entire circadian cycle).

### Tissue processing and cell counting

The specificity of the virally mediated intersectional genetics approach was confirmed, and quantification of eYFP-positive somata was conducted, via tissue processing and epifluorescence microscopy (microscope model DM2500, Leica). Tissue was collected immediately from all saline-injected mice after they were killed and, when possible, from KA-injected mice following on-demand testing. While the IHKA model is not traditionally considered a model of sudden unexpected death in epilepsy (SUDEP) per se, a number of animals died before the 6 month time point because of seizures. Brains from these mice were collected as soon as possible, but were only used to verify opsin expression and targeting. They were not used for cell quantification. All brains were drop fixed in 4% paraformaldehyde (PFA) and sectioned into 50 μm coronal sections via vibratome (model VT1000S, Leica) that were mounted with DAPI (VECTASHIELD Antifade Mounting Media with DAPI, catalog #H-1200, Vector Laboratories) as a 1-in-4 series. With the exception of tissue from one mouse that was collected too long postmortem, a lack of eYFP expression was assessed and confirmed in all opsin-negative tissue (*n* = 19 of 20 mice). Similarly, the expression of eYFP in opsin-positive tissue was confirmed from all but 3 opsin-positive mice (*n* = 22 of 25 mice); these 3 animals had unusable tissue.

**Figure 2.**
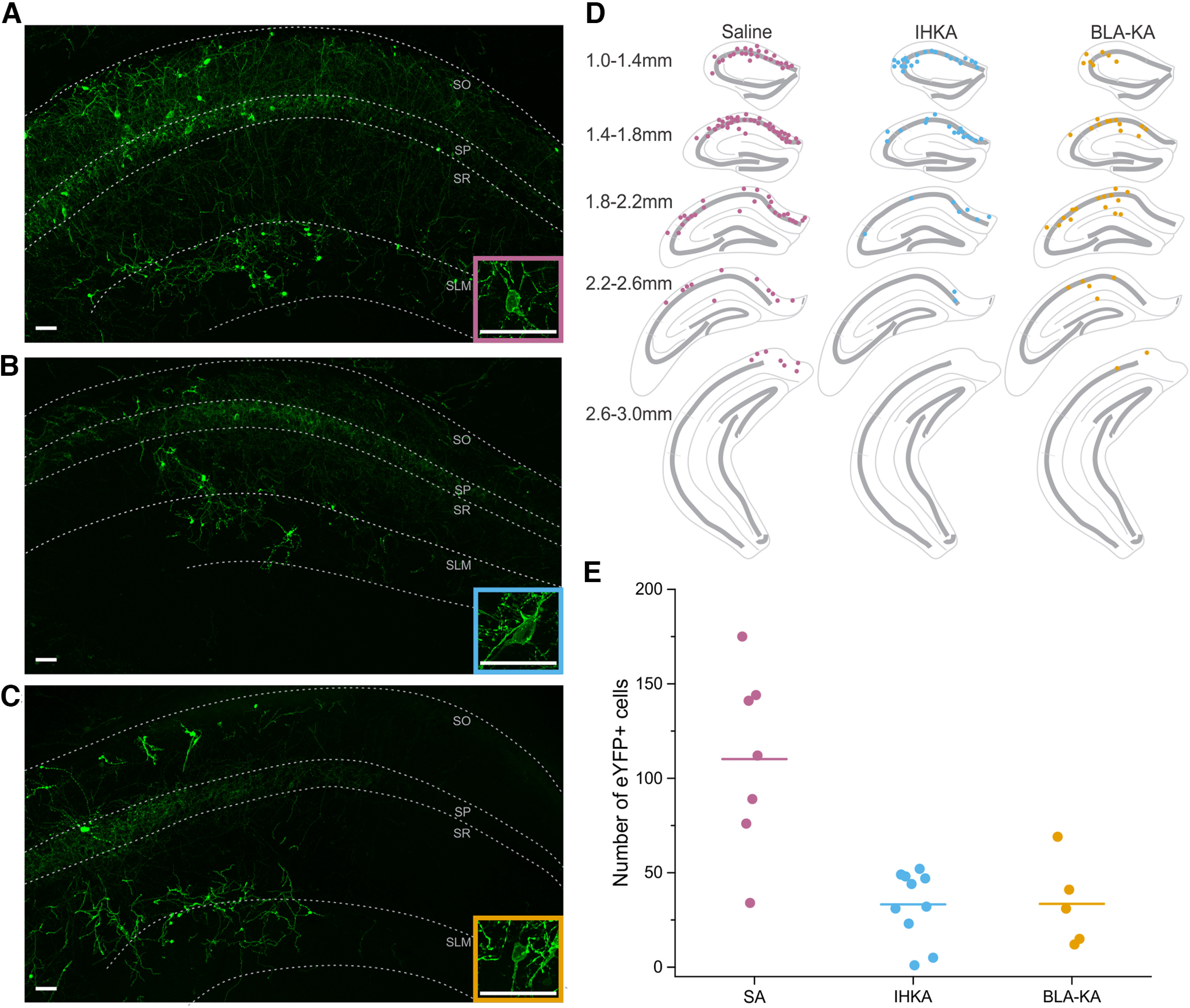
Substantial reduction of LINCs in epileptic tissue. eYFP-expressing somata were quantified from a 1-in-4 series of 50 μm hippocampal slices from virus-injected cNOS-fDLX mice. ***A–C***, Representative images of eYFP expression in CA1 ∼1.5 mm posterior to bregma. SO, Stratum oriens; SP, stratum pyramidale; SR, stratum radiatum; SLM, stratum lacunosum moleculare. Scale bars, 50 μm. ***A***, Widespread eYFP expression in a representative saline-injected mouse [overall cell count in this animal, 112 (equal to the median of all saline animals)]. Inset, Single eYFP-expressing cell from the same animal. ***B***, eYFP expression in an IHKA mouse (overall cell count in this animal, 48). Inset, Single eYFP-expressing cell from the same animal. ***C***, eYFP expression in an intra-amygdala kainate (BLA-KA) mouse (overall cell count in this animal, 41). Inset, Single eYFP-expressing cell from the same animal. ***D***, Example distributions of eYFP-positive somata along the hippocampal anterior–posterior axis in saline (left), IHKA (middle), and BLA-KA (right) mice (same animals as shown in ***A–C***). ***E***, The number of eYFP-positive cells was different between saline-injected, intrahippocampal kainate-injected, and intra-amygdala kainate-injected mice (*p* = 0.006, KW test, ANOVA). Control mice injected with saline have an average cell count of 110 ± 18.1 (SA, left). IHKA epileptic mice (IHKA, middle) had a significant reduction in the number of eYFP-positive cells, with an average cell count of 33 ± 5.8 (vs SA count, *p* = 0.004, MW test). Despite the site of initial insult being outside the hippocampus, BLA-KA animals (right) also had a significant reduction in the number of eYFP-positive cells in the hippocampus (average cell count, 33.6 ± 10.3 vs SA count, *p* = 0.015, MW test). Dots represent individual mouse values, and lines represent average group values (Table 1, a summary of statistical analyses).

Tissue that was collected immediately after mice were sacrificed was used for quantification of eYFP-expressing cells. This included 10 brains from opsin-positive IHKA mice (5 following 1 month post-KA intervention; 5 following 6 month post-KA intervention), and 7 from opsin-positive saline-injected mice (1–6 months postinjection; cell count not correlated with time post-saline injection; *p* = 0.38 Spearman’s correlation). The number of eYFP-positive somata in the hippocampal formation spanning from ∼1.0 to 3.8 mm posterior to bregma was counted manually in every fourth 50 μm section (14 50 μm sections total per animal) in all planes of focus at 20× magnification (model DM2500 microscope, Leica), in a manner similar to that in the study by Christenson Wick et al. (2019). Representative images in Figure 2 were obtained via confocal imaging at 10× and 40× magnification on an Olympus FluoView BX2 (University Imaging Center, University of Minnesota).

To quantify KA-induced changes in the number of PV-immunopositive cells, at 1 month postinjection, tissue was collected from six and five intrahippocampal KA-injected and SA-injected C57BL/6J mice, respectively. Brains were drop fixed in 4% PFA for 2 d immediately following mice being sacrificed and then sectioned into 50 μm sections. Matching quantification protocols for eYFP cells, a 1-in-4 series across the anterior–posterior extent of the hippocampus was used. To increase the penetration of antibodies, sections were suspended in 100% methanol for 3 h before free-floating PV immunostaining (1:1000; guinea pig anti-PV; GP72, Swant) in red. Sections were mounted with DAPI and imaged at 10× magnification (total internal reflection fluorescence (TIRF) scope, Zeiss; University Imaging Center, University of Minnesota). In total, 14 slices per animal spanning ∼1.0 to 3.8 mm posterior to bregma were used for the quantification of PV-immunopositive cells. Automated quantification of PV-immunopositive cells was conducted using background subtraction and additional thresholds, such as surface area, volume, and sphericity (Imaris software), followed by manual inspection of every slice and correction of cell counts, as necessary.

The approximate volume of tissue that would receive direct light stimulation was estimated using the Deisseroth Laboratory, Stanford University, Stanford, CA, online light transmission calculator (https://web.stanford.edu/group/dlab/cgi-bin/graph/chart.php). The optic fibers used had a NA of 0.39, a radius of 100 μm, and a flat cleaved tip. The average power measured *post hoc* from the fiber tips (4.9 mW) was used. An index of refraction for brain tissue of 1.35 (Binding et al., 2011) and an irradiance value of 1 mW/mm^2^ for ChR2 activation (Aravanis et al., 2007) were assumed. Accordingly, blue light delivery (473 nm) reached a depth of 0.9 mm. Given a half-angle of ∼16.8°, the maximum radius of the cone of light was ∼0.3 mm. Thus, with the optic fiber implanted at 2.2 mm posterior to bregma, light was directly delivered to a tissue volume expanding from ∼1.9 to 2.5 mm posterior to bregma along the anterior–posterior axis.

### Statistical analyses

After detection, seizure duration was determined offline using a combination of manual and automated processes (Krook-Magnuson et al., 2013, 2014a; Streng and Krook-Magnuson, 2020; Streng et al., 2021). Similar to online detection, user-defined aspects of ictal spiking were defined and used for automated analysis of the duration of seizures following detection. All automated detections were subsequently manually inspected, and at all steps in the process experimenters were blinded to light condition. For IHKA mice, a minimum of 90 seizures per light condition detected within 48 h were used for analysis; datasets with fewer seizures were excluded. Because of a lower rate of seizures in BLA-KA mice compared with IHKA mice, a dataset of 60 seizures per condition detected over 12 d was used for analysis. Two-sample Kolmogorov–Smirnov (KS) and two-tailed Mann–Whitney (MW) tests were conducted to compare postdetection seizure duration between light and no-light events at the individual animal level. Seizure duration was considered statistically different with light only if both KS and MW *p*-values were <0.05.

Reduction in seizure duration was calculated at the individual level as a percentage of the duration reduction and as the difference of the average seizure duration of light and no-light seizures in seconds.

To determine whether light delivery changed seizure duration at the group level within a genotype, a paired Wilcoxon test was used to compare average no-light and light seizure durations. Cohen’s *d* is one metric for assessing effect size, with a value of <0.2 considered to indicate a small effect size (Cohen, 1977). For each experimental group (e.g., opsin-positive, opsin-negative animals), Cohen’s *d* was calculated as the difference between average seizure duration with and without light delivery, divided by the pooled standard deviation (SD*p*; Eq. 1), as follows:

(1) $$\text{SD}p=\surd \left(\left({\text{SD}}_{\text{1}}{}^{\text{2}}+{\text{SD}}_{\text{2}}{}^{\text{2}}\right)/\text{2}\right),$$

where SD_1_ and SD_2_ are the SDs of each group.

For additional within-genotype group analysis, 90–100 events per condition were randomly sampled (via MATLAB) from each animal and used to create summative histograms to compare light and no-light seizure duration distributions via a two-sample KS test.

To assess the change in seizure reduction or duration over time, or with the power of light delivered, a Spearman’s correlation was conducted.

To compare the percentage of reduction and the magnitude of seizure reduction in seconds between genotypes, a two-tailed MW test was used. To determine whether light-mediated reduction in seizure duration varied across genotype and either sex or stimulation parameter settings, a two-way ANOVA was used. To compare the proportion of animals with a statistically significant reduction in seizure duration with light across genotypes, the Pearson’s χ^2^ test was used.

To compare the number of eYFP-positive cells among saline, IHKA, and BLA-KA tissue, a Kruskal–Wallis ANOVA was used. To further compare the number of eYFP-positive cells between saline-injected and KA-injected tissue (IHKA or BLA-KA), a two-tailed MW test was used. To compare the number of PV-immunopositive cells between saline and IHKA tissue, a two-tailed MW test was used. To compare the number of PV-immunopositive cells and eYFP-positive cells in IHKA within the volume of tissue receiving direct light stimulation, a two-tailed MW test was used. To assess eYFP cell count over time in saline-injected tissue, a Spearman’s correlation was conducted.

Statistical analyses were conducted using MATLAB (versions 2016-2021b) and OriginPro software (2016). For all statistical tests, a *p*-value of <0.05 was considered statistically significant. All values are reported as the mean ± SEM, unless noted otherwise.

## Results

### On-demand activation of LINCs does not substantially influence seizure activity 1 month after epilepsy induction

LINCs have been identified recently as a powerful and widespread source of inhibition in the hippocampus (Christenson Wick et al., 2019). We therefore examined whether activating LINCs could provide seizure control using on-demand optogenetics to selectively activate LINCs at the time of hippocampal seizures. As illustrated in Figure 1, *A* and *B*, we used a viral intersectional genetics approach to label and express ChR2 in LINCs in CA1 of the hippocampus. We then induced epilepsy via intrahippocampal KA injection and implanted a recording electrode (for seizure detection) and an optic fiber (for light delivery). Online seizure detection triggered light activation of LINCs for 50% of detected events at random, providing a no-stimulation internal control (Fig. 1*C*, example trace). Opsin-negative animals served as an additional control and underwent identical experimental procedures. We first examined the impact of light delivery at ∼7 Hz with a 50 ms pulse width for 30 s; these stimulation parameters were chosen as they have previously been demonstrated to be effective for PV interneuron-mediated on-demand optogenetic seizure suppression in this same model of epilepsy (Krook-Magnuson et al., 2013).

At the individual animal level, the majority of opsin-positive animals showed no significant effect of light delivery; 13 of 21 (∼62%) opsin-positive animals had no significant effect of light delivery. In the remaining eight opsin-positive animals, light delivery statistically reduced seizure duration compared with control no-light seizures (*p* < 0.05 for KS and MW tests in these animals). However, statistically significant seizure reduction in these animals did not correspond with sizeable suppression, as illustrated in Figure 1*D*, which shows seizure duration distributions for an example animal with a statistically significant reduction in seizure duration with light; note the lackluster effect. In this animal, seizure durations were reduced by only 12.0%, corresponding to a duration reduction of just 2.5 s. The maximum observed percentage of reduction across animals was only 24.7%, and this corresponded to a reduction in seconds of just 5.7 s (Fig. 1*Eii*,*Fii*). This indicates that even in individual animals where a statistically significant seizure duration reduction was achieved, effects remained small.

Further underscoring the lack of successful intervention in opsin-positive animals, changes in seizure durations reached statistical significance in a similar proportion of opsin-negative animals (5 of 14 for opsin-negative animals vs 8 of 21 for opsin-positive animals, *p* = 0.13, Pearson’s χ^2^ test), suggesting that the small, sometimes statistically significant, effects observed with opsin-positive animals are unlikely to reflect meaningful effects of LINC activation.

Across opsin-positive animals with 7 Hz stimulation, there was an average 11.3 ± 1.8% decrease in seizure duration (Fig. 1*Fi*), corresponding to only a 2.0 ± 0.41 s reduction in average seizure durations (Fig. 1*Fii*), and a small effect size of light delivery (Cohen’s *d* = 0.14). These reductions were not significantly greater than those observed in opsin-negative animals (Fig. 1*F*; 8.9 ± 2.3% decrease or 2.0 ± 0.53 s decrease in seizure duration in opsin-negative animals; opsin-positive vs opsin-negatives: *p* = 0.39, MW test for percentage reduction; *p* = 0.97, MW for reduction in seconds), which also demonstrated a small effect of light delivery (Cohen’s *d* = 0.12). No apparent sex differences were observed across genotypes for either measurement of light-mediated reduction (percentage reduction: genotype, *p* = 0.34; sex, *p* = 0.07; interaction, *p* = 0.39; reduction in seconds: genotype, *p* = 0.90; sex, *p* = 0.20; interaction, *p* = 0.55; two-way ANOVAs).

### Using a different stimulation pattern does not improve outcomes

To test whether the lack of LINC-mediated influence on seizure activity was possibly because of the pattern of light stimulation, we tested an additional set of stimulation parameters previously used to successfully inhibit seizures through on-demand optogenetics (Krook-Magnuson et al., 2014b). Specifically, we tested 1,000 ms long pulses delivered for 3 s. At the group level, effects on seizure duration were small and not different between genotypes (Fig. 1*G*; opsin-positive: 8.9 ± 2.2%, 1.7 ± 0.5 s reduction; opsin-negative: 9.3 ± 2.6%, 1.6 ± 0.4 s reduction; opsin-positive vs opsin-negative: *p* = 0.94, MW test for percentage reduction; *p* = 0.69, MW test for reduction in seconds; Cohen’s *d* opsin-positive, 0.15; opsin-negative, 0.09). Overall, across genotypes the average and range of light-mediated seizure reduction with long-pulse stimulation was similar to 7 Hz stimulation (Fig. 1, compare *F*, *G*; percentage reduction: opsin-positive vs opsin-negative, *p* = 0.66; long-pulse vs 7 Hz stimulation, *p* = 0.66; interaction, *p* = 0.53; reduction in seconds: opsin-positive vs opsin-negative, *p* = 0.99; long-pulse vs 7 Hz stimulation, *p* = 0.51; interaction, *p* = 0.93; two-way ANOVAs). Therefore, regardless of the stimulation parameters used, there is little impact of LINC activation on seizures; at 1 month post-KA injection, LINC activation did not meaningfully influence seizure activity, despite LINCs providing widespread inhibition in healthy tissue (Christenson Wick et al., 2019).

### A substantial reduction in the number of LINCs is observed in epileptic mice

Given that LINCs are a powerful source of inhibition in healthy tissue (Christenson Wick et al., 2019), we were surprised that LINC-mediated seizure suppression provided so little seizure control. Given the large circuit changes that can occur in temporal lobe epilepsy, we examined whether LINCs persisted in the epileptic tissue. Cell counts were conducted from 10 opsin-positive animals that received intervention, including 5 animals that had received on-demand optogenetic intervention 1 month post-KA injection, and 5 that had received on-demand optogenetic intervention up to 6 months post-KA injection (see next section). For comparison, cell counts were also conducted for saline-injected opsin-positive animals, with tissue collected 1–6 months post-saline injection. Robust labeling of LINCs was seen in saline-injected control animals (Fig. 2*A*,*E*), extending along the anterior–posterior extent of the hippocampus (Fig. 2*D*), as previously reported (Christenson Wick et al., 2019). A dramatic reduction in eYFP-labeled cells was observed in epileptic animals (Fig. 2*B*,*D*,*E*; *p* = 0.004, MW test), indicating that LINCs are vulnerable in epileptic tissue. Note that viral labeling of LINCs occurred before KA injection, and therefore the lower number of labeled LINCs is unlikely to reflect differences in initial transduction. This substantial loss of LINCs in epileptic mice may contribute to the inability to inhibit seizures via on-demand optogenetic activation of LINCs.

Given that on-demand optogenetic activation of PV-expressing interneurons has previously been shown to inhibit hippocampal seizures in the IHKA model (Krook-Magnuson et al., 2013), we additionally examined the vulnerability of PV cells in our hands. While PV cells make up <5% of neurons in the hippocampus (Woodson et al., 1989; Freund and Buzsáki, 1996), PV-immunopositive cell numbers in saline-injected animals far outnumbered eYFP-labeled LINCs (1800 ± 168 PV-immunopositive cells counted over 14 sections; one in four series). In IHKA animals, the number of PV-immunopositive cells was significantly reduced (825 ± 61 cells; *p* = 0.008 vs saline controls, MW test; 54% reduction). This suggests that both PV cells and LINCs are vulnerable populations.

While a number of additional factors will play critical roles in the ability of light delivery to impact seizures, we directly compared the relative numbers of PV-immunopositive cells and eYFP-ChR2-expressing LINCs in the vicinity of light delivery in IHKA animals. Ignoring axonal activation, these cells are those that would be activated by light delivery. Given the calculated potential spread of light, we examined three sections centered around the location of the light source. In these sections, we counted 231 ± 34 PV-immunopositive cells in saline controls, which was reduced to 49 ± 17 cells in IHKA animals (representing a 79% reduction in cell numbers). In contrast, even in saline-injected controls, we found only 38 ± 6 eYFP-labeled LINCs in these three sections, and this was further reduced to only 9 ± 2 eYFP-labeled cells in IHKA tissue. While this represents a similar percentage reduction (76% decrease), the result is a stark contrast in the raw number of remaining cells (nearly 50 vs <10; *p*= 0.002 MW). Therefore, while both populations of interneurons are impacted in the IHKA model, the remaining number of targetable cells is much higher for PV interneurons than LINCs.

### Moving the site of initial insult does not change outcomes

The noted LINC cell loss could have resulted from epilepsy itself (e.g., seizures) or could have been a consequence of injecting KA directly into the hippocampus. To begin to examine whether LINC loss is specific to the intrahippocampal kainate model, and correspondingly whether LINC activation might be more efficacious in a different model, we turned to the intra-amygdala model of temporal lobe epilepsy. In this model, KA is not injected into the hippocampus, but is instead injected into the basolateral amygdala (BLA-KA; Ben-Ari et al., 1980; Mouri et al., 2008), and spontaneous hippocampal electrographic and behavioral seizures are observed during the chronic phase (Li et al., 2008). We tested on-demand optogenetic activation of LINCs in one BLA-KA animal but found no significant change in seizure duration (*p* = 0.89, KS test). In fact, we saw a slight (5.8%) increase in average seizure duration with light in this animal. This suggests that the lack of LINC-mediated seizure control is not model specific. Additionally, despite a different site of initial direct injury, we found that there was still a significant reduction in the number of eYFP-positive cells in BLA-KA animals compared with saline-injected controls (Fig. 2*E*, *p* = 0.015, MW test; Fig. 2*C*,*D*, animal illustrated is the animal that received on-demand intervention). Thus, regardless of the site of KA injection, a large reduction in the number of LINCs was observed in epileptic animals, which may impact the potential for LINC-mediated seizure suppression.

### Lack of LINC-mediated seizure suppression up to 6 months post-epilepsy induction

Axonal sprouting of inhibitory neurons in epilepsy has been observed (Zhang et al., 2009; Peng et al., 2013; Soussi et al., 2014), including sprouting that occurs 4–6 months post-KA injection (Christenson Wick et al., 2017). As axonal sprouting at later time points post-KA injection could potentially compensate for inhibitory cell loss and allow more meaningful seizure control, we examined whether LINC-mediated influence on seizures would change over time. We tested animals with 7 Hz and long-pulse stimulation, as done at the 1 month post-KA injection time point (described above), each month for up to 6 months post-KA injection.

**Figure 3.**
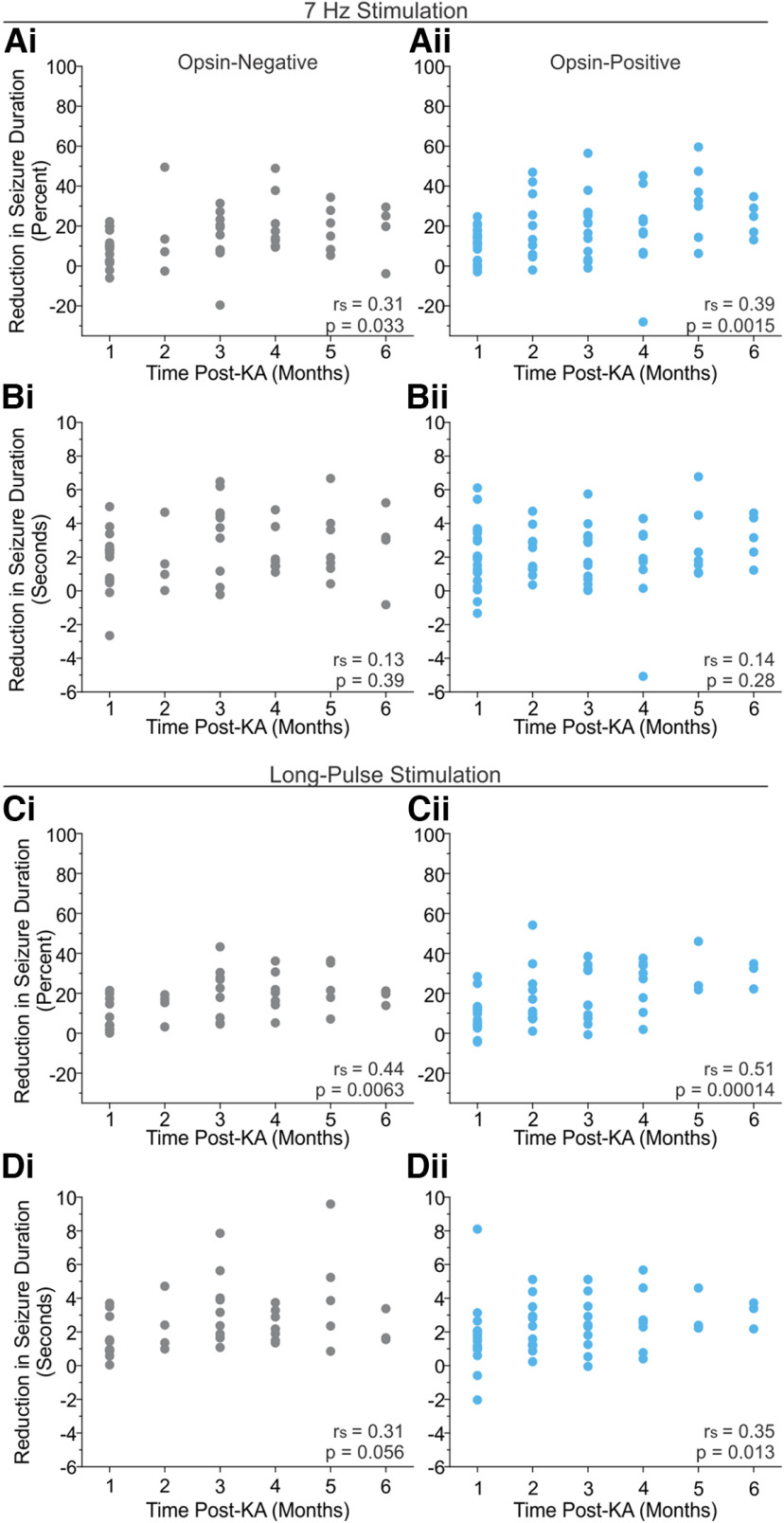
LINC activation does not substantially influence seizure duration for up to 6 months post-epilepsy induction. Reduction of seizure duration with light stimulation over time since the injection of KA to induce epilepsy. Note that, when possible, mice were tested at multiple time points, but some mice were excluded from some time points because of SUDEP or an insufficient number of events for analysis. ***A***, ***B***, The percentage reduction (***A***), but not the reduction in seconds (***B***), of seizure duration with 7 Hz light stimulation slightly increases over time. Light-mediated seizure suppression remains modest throughout. At 6 months post-KA, the maximum reductions are only 34.7% or 4.6 s, and at no point are effects notably larger in opsin-positive animals. ***C***, ***D***, Similar results occur with long-pulse light stimulation, with little change observed over time. At 6 months post-KA, the maximum reduction in seizure durations are only 34.8% or 3.7 s. The *p*-values correspond to Spearman’s correlation (*r_s_* = Spearman’s Rho; Table 1, summary of statistical analyses).

In opsin-positive animals, the average percentage reduction in seizure duration slightly increased over time with 7 Hz stimulation of LINCs (Fig. 3*Aii*, Table 1; *p* = 0.0015, *r*_s_ = 0.39, Spearman’s ρ correlation). However, the percentage reduction remained small at all time points (Fig. 3). Moreover, when the reduction in seizure duration was measured in seconds, there was no such correlation with time since epilepsy induction (Fig. 3*Bii*; *p* = 0.28, Spearman’s correlation). This suggests that the change in percentage reduction may reflect a difference in overall seizure durations (average seizure duration without light vs time: *p* = 1.95 * 10^−5^, *r*_s_ = −0.50; average seizure duration with light delivery versus time: *p* = 2.56 * 10^−5^, *r*_s_ = −0.50), rather than a change in the effectiveness of light per se. Note that not all animals have data from all time points. Importantly, LINC-mediated reduction in seizure duration remained modest even at 6 months: the maximum reduction in seizure duration with 7 Hz stimulation in opsin-positive animals was 34.7%, but this corresponded to only a 3.2 s reduction in average seizure duration in this animal. The maximum reduction in seconds was similarly modest, and was only 4.6 s (corresponding to 17.0%).

Further underscoring that no meaningful seizure control was ever achieved in opsin-positive animals, a similar slight increase in percentage reduction of seizure durations over time post-KA injection was also observed with 7 Hz stimulation in opsin-negative animals (Fig. 3*Ai*; *p* = 0.033, *r*_s_ = 0.31, Spearman’s correlation). This was accompanied by no change across time in absolute reduction in seconds (Fig. 3*Bi*; *p* = 0.39, *r*_s_ = 0.13) nor average seizure duration with or without intervention (average seizure duration without light: *p* = 0.12, *r*_s_ = −0.23; average seizure duration with light-delivery: *p* = 0.078, *r*_s_ = −0.26 Spearman’s correlation).

Similar to 7 Hz stimulation, long-pulse stimulation showed a slight increase in percentage reduction over time, but this was again very modest and was observed in both opsin-negative and opsin-positive animals (Fig. 3*C*). A very slight increase over time was observed for seizure reduction measured in seconds in opsin-positive animals, but the impact of light remained modest and was never significantly greater than that seen in opsin-negative animals (Fig. 3*D*). Altogether, these results support the idea that, even at later time points post-KA injection, LINC activation is unable to substantially influence seizure activity.

## Discussion

In healthy tissue, hippocampal LINCs provide widespread inhibition mediated by both GABA_A_ and GABA_B_ postsynaptic receptors (Christenson Wick et al., 2019). We therefore hypothesized that they may be strong targets for seizure control using on-demand optogenetics. However, regardless of the stimulation parameters chosen, or the time post-KA injection examined, no strong seizure control was ever achieved through activation of LINCs. We noted a substantial reduction in the number of LINCs in epileptic tissue, including when the site of initial insult was not in the hippocampus itself. This suggests that LINCs are a vulnerable cell population in epilepsy, which may contribute to the lack of efficacy when targeting LINCs for seizure control.

Optogenetics has been extensively used in the study of epilepsy (for review, see Christenson Wick and Krook-Magnuson, 2018; Cela and Sjöström, 2019; Walker and Kullmann, 2020). Previous work using on-demand optogenetics, with a variety of target strategies, has successfully inhibited seizures in the IHKA model of TLE (Krook-Magnuson et al., 2013, 2014b, 2015; Bui et al., 2018; Streng and Krook-Magnuson, 2020; Chen et al., 2021; Hristova et al., 2021; Streng et al., 2021). This includes work targeting populations in the hippocampus (Krook-Magnuson et al., 2013, 2015; Bui et al., 2018; Chen et al., 2021), indicating that this can be a successful approach—if the correct populations are manipulated in the correct way. On-demand optogenetics directly inhibiting principal cells in the hippocampus provided strong inhibition of electrographic seizures, with noted percentage reductions ranging from ∼70% (Krook-Magnuson et al., 2013) to 75% (Krook-Magnuson et al., 2015). Previous work also found that on-demand optogenetic excitation of a different population of interneurons, PV-expressing interneurons, was able to inhibit seizures, although with less success than direct inhibition of principal hippocampal cells (Krook-Magnuson et al., 2013; Chen et al., 2021). The role of PV interneurons in seizures is complicated and depends on a range of factors, including the health of the tissue, whether seizures are acutely induced or spontaneously arising in epileptic tissue (and if induced, how and where), developmental stage and epilepsy type, and the relative timing of activation (Ellender et al., 2014; Sessolo et al., 2015; Assaf and Schiller, 2016; Lu et al., 2016; Shiri et al., 2016, 2017; Wang et al., 2017, 2022; Favero et al., 2018; Maguire, 2018; Lévesque et al., 2019; Magloire et al., 2019; Krook-Magnuson, 2020; Chen et al., 2021; Dudok et al., 2022; Kaneko et al., 2022).

Most relevant to the work presented here, however, is work using on-demand optogenetic excitation of PV interneurons in the hippocampus with the IHKA mouse model, which noted significant seizure inhibition, with an average percentage seizure duration reduction across animals of ∼35–50% (Krook-Magnuson et al., 2013, 2014b; Chen et al., 2021). Notably, this was significantly greater than opsin-negative controls in those studies and is markedly higher than the average percentage seizure reduction achieved here with on-demand optogenetic activation of LINCs. We found that LINC stimulation—with the same protocols used to successfully inhibit seizures via activation of PV cells—produced only an average 8.9–11% seizure duration reduction; these values were not significantly greater than opsin-negative controls. We initially hypothesized that on-demand optogenetic activation of LINCs would outperform seizure inhibition achieved by on-demand optogenetic activation of PV cells, as—in healthy tissue—LINCs provide strong inhibition to both deep and superficial CA1 pyramidal cells, and inhibition via both GABA_A_ and GABA_B_ mechanisms (Christenson Wick et al., 2019). In contrast, PV cells preferentially target deep pyramidal cells and primarily activate just GABA_A_ receptors (Armstrong and Soltesz, 2012; Lee et al., 2014), which would be sensitive to a collapse in the chloride gradient (Dzhala and Staley, 2003; Cossart et al., 2005; Trevelyan and Schevon, 2013; Wang et al., 2017). However, the activation of LINCs failed to outperform the activation of PV cells and, indeed, failed to provide any meaningful seizure inhibition.

A number of factors may have contributed to the failure of on-demand optogenetic activation of LINCs to inhibit seizures. First and foremost, we noted a substantial decrease in the number of LINCs in epileptic tissue, resulting in very few remaining eYFP-ChR2-expressing cells. This, obviously, reduces the number of LINCs that can be activated by our on-demand optogenetic approach, limiting the potential impact of LINC activation. PV cells can also be lost in epilepsy (Maglóczky and Freund, 2005; Kuruba et al., 2011), including in the IHKA model of TLE (Marx et al., 2013). We found that the average number of PV-immunopositive cells in IHKA animals was reduced by 54% across the entire anterior–posterior axis, and by 79% specifically at the location light was delivered in this study. This percentage reduction at the site of light delivery was similar to the percentage reduction observed in eYFP-ChR2-labeled LINCs. How can PV cells then remain a viable target? First, the absolute number of remaining PV cells at the location of light delivery in this study was approximately five times that of labeled LINCs. Therefore, there would be substantially more cells available to potentially provide seizure control. Additionally, any axons in the path of light could additionally be activated. This is especially relevant, as it has been noted that inhibitory neurons can undergo axonal sprouting in epilepsy (Zhang et al., 2009; Peng et al., 2013; Soussi et al., 2014). Importantly, this includes sprouting of the axons of PV cells (Christenson Wick et al., 2017). In the IHKA model of TLE, long-range axonal sprouting from PV cells was noted 4–6 months post-KA injection (Christenson Wick et al., 2017). Axonal sprouting of surviving neurons may help compensate for cell loss. However, for LINCs, if axonal sprouting occurs, it appears to be insufficient to meaningfully impact outcomes: even at late time points, up to a full 6 months post-KA injection, on-demand optogenetic excitation of LINCs failed to provide meaningful seizure suppression (Fig. 3). Even without an epileptic insult, LINCs are a relatively sparse hippocampal neuronal population (Christenson Wick et al., 2019; compare also our saline cell counts), and sprouting of the axons of LINCs may be insufficient to overcome a further reduction in their numbers. Additionally, our experimental methods may not have allowed full capitalization of sprouting: most previous work targeting PV neurons in the IHKA model used a transgenic approach (Krook-Magnuson et al., 2013, 2014b), resulting in all PV neurons expressing channelrhodopsin. Therefore, any PV cell now innervating the illuminated area would be able to contribute to seizure inhibition. In contrast to target LINCs, we had to take an intersectional genetic, viral vector-based approach. While we transfected LINCs for channelrhodopsin expression before the epileptogenic insult (to ensure robust transfection), and while we saw good viral spread with our approach (Fig. 2), areas more remote (including the contralateral hippocampus) would not be able to contribute to seizure suppression in the same manner. An additional difference between PV cells and LINCs is also worth noting: in addition to broadly inhibiting CA1 pyramidal cells, in healthy tissue LINCs also provide inhibition to inhibitory neurons. This could theoretically counteract the beneficial effects of inhibition they provide to pyramidal cells. However, many seizure medications increase inhibition broadly with at least some degree of success (Greenfield, 2013; Holtkamp, 2018; Kotloski and Gidal, 2022). Given the dramatic loss of LINCs in epilepsy, a more likely interpretation is insufficient inhibition, rather than poorly directed inhibition. However, a recent study did find greater on-demand optogenetic seizure control when targeting only hippocampal PV cells compared with broadly targeting hippocampal inhibitory interneurons (via DLX6/5; Chen et al., 2021).

We examined two different stimulation protocols (and neither worked), and we used light stimulation parameters that were previously successful when targeting other neuronal populations (Krook-Magnuson et al., 2013, 2014b). However, we cannot rule out that different experimental methods, including different light delivery paradigms, might be able to achieve greater seizure suppression via LINC activation. Previous work in healthy tissue demonstrated that a train of 50 ms light pulses produces repeated LINC-mediated postsynaptic inhibition, although, notably, there is substantial fatigue after the first light pulse (Christenson Wick et al., 2019).

Additionally, activation of LINCs *in vivo* revealed that 7 Hz stimulation alters hippocampal theta oscillations for several seconds, although, again, there was a decline in amplitude within the first 500 ms of light onset (Christenson Wick et al., 2019). Altogether, this provides evidence that LINCs can alter network activity over several seconds, with protocols similar to those used here, at least in healthy tissue. However, the substantial decrease in the amplitude of LINC-mediated inhibition over the course of the stimulation train may contribute to the poor seizure suppression observed. While it is unclear whether such fatigue could be overcome to allow for more successful intervention strategies, future work could potentially apply methods such as Bayesian optimization to examine whether LINC-mediated seizure suppression could be improved with different stimulation parameters (Stieve et al., 2023). Regardless of why LINC activation was unable to provide meaningful seizure control, and regardless of whether slightly better inhibition could have been achieved through different experimental methods, our results strongly indicate that on-demand activation of LINCs for seizure control is less robust than other approaches already demonstrated to be effective.

Our results also suggest that LINCs are a sensitive population, undergoing substantial cell loss in epileptic tissue. Importantly, moving the site of kainate injection did not rescue LINC numbers. Hippocampal cell death in BLA-KA mice is not atypical—pathologic changes in the hippocampus, including sclerosis marked by pyramidal cell loss, is observed following intra-amygdala kainate injections (Araki et al., 2002; Mouri et al., 2008). Preventing status epilepticus through the administration of diazepam before intra-amygdala KA injection did not alter the amygdalar lesion at the site of injection, but did result in a lack of hippocampal sclerosis (Ben-Ari and Lagowska, 1978). Thus, hippocampal sclerosis in BLA-KA mice is attributed to status and the propagation of seizures, further suggesting that seizures, rather than the (location of the) initial insult per se, caused LINC cell loss. As noted above, in healthy tissue, LINCs provide widespread inhibition within the hippocampus, and their loss therefore may contribute to phenotypes in epilepsy, including cognitive and affective phenotypes (Muldoon et al., 2015; Moxon et al., 2019; Shuman et al., 2020). LINCs also have long-range projections to areas outside the hippocampus, including the tenia tecta, the hypothalamus, and the medial septum (Christenson Wick et al., 2019). LINCs may play an important role in coordinating the activity between the hippocampus and these regions (Christenson Wick et al., 2019), and the loss of LINCs in epilepsy may also contribute to comorbidities and circuit dysfunction through the loss of this interregional communication. LINCs remain a poorly understood cell population, and a greater understanding of their role in healthy physiology and in epilepsy will be an important goal for future research.

There are a few important experimental aspects to this study to further consider. First, we examined the ability of LINCs to inhibit seizures in a model of chronic temporal lobe epilepsy that models not only seizures, but also hippocampus sclerosis (Suzuki et al., 1995; Bouilleret et al., 2000). Different results may have been achieved if we had instead used, for example, acute seizure induction in healthy tissue. Such results would then have failed to translate to situations with the (at times substantial) circuit changes that can happen in chronic temporal lobe epilepsy (de Lanerolle et al., 1992; Berg et al., 2009; Twele et al., 2016). The seizure model used—and subsequent differences in cytoarchitecture and pathology—has been associated with conflicting results across studies targeting other hippocampal inhibitory and excitatory cell populations. For example, the activation of mossy cells in the dentate gyrus was proconvulsant in an acute seizure model (Botterill et al., 2019), but anticonvulsant in the IHKA chronic epilepsy model (Bui et al., 2018). Second, even in the same model of temporal lobe epilepsy, there is considerable animal to animal variability. This highlights the importance of within-animal comparisons, but also the importance of considering subject dropout in results. Across time, animals that remained in our study showed, on average, a decrease in seizure durations with time. This resulted in an increase in the percentage of seizure reduction in time despite a relatively stable absolute reduction (in seconds) of seizure duration. Importantly, some of the animals (presumably those with more severe initial epilepsy) were lost to SUDEP over the course of our experiments, which likely contributed to this observed change in seizure durations over time at the group level. A final important aspect to consider is that our experiments were well controlled, with both internal no-light controls and group comparisons to opsin-negative animals that received otherwise identical experimental procedures. The use of opsin-negative animals was critical, as it helped contextualize the small, but at times statistically significant, effects we did observe with light delivery. While some of this may be because of chance, and while effects in opsin-negative animals were always very small, it provided a key point of reference; without this context, we may have overinterpreted the occasional small, but significant, effect in opsin-positive animals.

Although light delivery always resulted in at most small effect sizes in opsin-negative animals, that any significant effect was observed is surprising. What may be driving this small, yet at times significant, effect? Light delivery alone can have impacts on neuronal systems through a variety of mechanisms. Light-induced tissue heating is one such possibility (Arias-Gil et al., 2016; Gysbrechts et al., 2016; Shin et al., 2016; Peixoto et al., 2020; Acharya et al., 2021). In the present study, following data collection, light power measured through the tip of the implanted optic fiber was an average of 4.9 ± 0.4 mW. While this is not generally considered a high level of light for optogenetic studies, this amount of light could, in theory, have a small influence on neural activity via tissue heating (Arias-Gil et al., 2016; Acharya et al., 2021). However, we found that light power was not correlated with the percentage reduction in seizure duration with light (*p* = 0.60, Spearman’s correlation), making it unlikely that tissue heating was the critical factor for the observed light-mediated effects. Another potential impact of light delivery alone is the alteration of neurologic activity via the visual pathway (Iaccarino et al., 2016; Jones et al., 2019; Chan et al., 2021). While optical connections were covered to mitigate external light delivery with stimulation, it did not prevent all of it. Therefore, a visual impact theoretically may have contributed to shorter average seizure durations with light. However, if mediated by a visual effect, it does not appear to be impacted by the frequency of light pulses, as the effects were similar between the two sets of stimulation parameters (opsin-negative percentage reduction, 7 vs 1 Hz; *p* = 0.44, MW test). Another potential source of light-mediated effects in opsin-negative animals is light-mediated alterations in blood flow (especially with blue light; Rungta et al., 2017). If light-mediated alterations in blood flow can alter seizures, this could be an interesting avenue for future investigations. However, the very small size of the noted effects is worth remembering. Any future investigations attempting to build on the finding would need to substantially increase the effect to have a meaningful impact on seizures. While any off-target effects of light were not large enough to be clinically relevant in our experiments, they are still an important experimental factor to consider when designing and interpreting results. Consideration of all of these factors led us to the ultimate conclusion that on-demand optogenetic excitation of LINCs cannot provide robust seizure inhibition.

LINCs are an understudied hippocampal neuronal population, with a number of unique and exciting features (Christenson Wick et al., 2019). While they do not appear to be a strong candidate target for seizure interventions, a better understanding of their role in healthy physiology, and in turn the impact of LINC loss in epilepsy, will be important areas for future investigation.

## Synthesis

Reviewing Editor: Viji Santhakumar, University of California Riverside Decisions are customarily a result of the Reviewing Editor and the peer reviewers coming together and discussing their recommendations until a consensus is reached. When revisions are invited, a fact-based synthesis statement explaining their decision and outlining what is needed to prepare a revision will be listed below. The following reviewer(s) agreed to reveal their identity: Csaba Varga, MacKenzie Howard.

The reviewers are in agreement that the authors have addressed all major issues raised in the initial review and that the manuscript is much stronger.

Some minor concerns remain that could be readily addressed simple analysis and/or edits to the manuscript.

Concerns regarding the change in seizure duration in opsin-negative animals are not fully addressed. In the results section, the authors state “However, statistically significant seizure reduction in these animals did not correspond with meaningful suppression; effect sizes were always small.” This change in duration still corresponds to a statistically significant ∼ 15-20% which is not trivial. Was effect size explicitly calculated? This needs to be included. It is interesting that, in the longer study, none of the mice exhibited increases in seizure duration suggesting that light may be reducing duration. While the reviewers are comfortable with the data as is, there needs to be a quantitative basis for indicating that the effect size is “small”. In this regard, statistical comparison of seizure duration times in opsin-negative mice at the later time points compared with the one-month post-KA time point could reveal if seizures are getting a little shorter.

Two things worth clarifying in the introduction:

There’s a great discussion of the diversity of interneuron subtypes. This then transitions into discussion of PV interneuron modulation. It should be mentioned that PV expression encompasses multiple subtypes of interneurons, and this in and of itself makes the use of biochemical markers for labeling and for activating for seizure suppression, complicated.

“Optogenetic activation of LINCs in hippocampal slices results in postsynaptic inhibition of 80% of all cell types in CA1...” Does this mean 80% of cell types, or 80% of cells? Does this include inhibition of other interneurons, or inhibition of other LINCs?

## References

[B1] Acharya AR, Vandekerckhove B, Larsen LE, Delbeke J, Wadman WJ, Vonck K, Carette E, Meurs A, Vanfleteren J, Boon P, Missinne J, Raedt R (2021) *In vivo* blue light illumination for optogenetic inhibition: effect on local temperature and excitability of the rat hippocampus. J Neural Eng 18:066038. 10.1088/1741-2552/ac3ef4 34951406

[B2] Alexander A, Maroso M, Soltesz I (2016) Organization and control of epileptic circuits in temporal lobe epilepsy. Prog Brain Res 226:127–154. 10.1016/bs.pbr.2016.04.007 27323941 PMC5140277

[B3] Alfonsa H, Merricks EM, Codadu NK, Cunningham MO, Deisseroth K, Racca C, Trevelyan AJ (2015) The contribution of raised intraneuronal chloride to epileptic network activity. J Neurosci 35:7715–7726. 10.1523/JNEUROSCI.4105-14.2015 25995461 PMC4438123

[B4] Araki T, Simon RP, Taki W, Lan JQ, Henshall DC (2002) Characterization of neuronal death induced by focally evoked limbic seizures in the C57BL/6 mouse. J Neurosci Res 69:614–621. 10.1002/jnr.10356 12210827

[B5] Aravanis AM, Wang LP, Zhang F, Meltzer LA, Mogri MZ, Schneider MB, Deisseroth K (2007) An optical neural interface: in vivo control of rodent motor cortex with integrated fiberoptic and optogenetic technology. J Neural Eng 4:S143–S156. 10.1088/1741-2560/4/3/S02 17873414

[B6] Arias-Gil G, Ohl FW, Takagaki K, Lippert MT (2016) Measurement, modeling, and prediction of temperature rise due to optogenetic brain stimulation. Neurophotonics 3:045007. 10.1117/1.NPh.3.4.045007 27981063 PMC5129112

[B7] Armstrong C, Soltesz I (2012) Basket cell dichotomy in microcircuit function. J Physiol 590:683–694. 10.1113/jphysiol.2011.223669 22199164 PMC3381302

[B8] Armstrong C, Krook-Magnuson E, Soltesz I (2012) Neurogliaform and Ivy cells: a major family of nNOS expressing GABAergic neurons. Front Neural Circuits 6:23. 22623913 10.3389/fncir.2012.00023 PMC3353154

[B9] Armstrong C, Krook-Magnuson E, Oijala M, Soltesz I (2013) Closed-loop optogenetic intervention in mice. Nat Protoc 8:1475–1493. 10.1038/nprot.2013.080 23845961 PMC3988315

[B10] Assaf F, Schiller Y (2016) The antiepileptic and ictogenic effects of optogenetic neurostimulation of PV-expressing interneurons. J Neurophysiol 116:1694–1704. 10.1152/jn.00744.2015 27486107 PMC5144715

[B11] Ben-Ari Y, Lagowska J (1978) Action épileptogène induite par des injections intraamygdaliennes d'acide kainique. C R Acad Hebd Seances Acad Sci D 287:813–816. 103652

[B12] Ben-Ari Y, Tremblay E, Ottersen OP, Meldrum BS (1980) The role of epileptic activity in the hippocampus and “remote” cerebral lesions induced by kainic acid. Brain Res 191:79–97. 10.1016/0006-8993(80)90316-9 7378761

[B13] Benassi PB (1962) Experimental research on the anticonvulsant properties of 1-glutamine, 1-asparagine, gamma-aminobutyric acid and gamma-amino-beta-hydroxybutyric acid. Riv Sper Freniatr Med Leg Alien Ment 86:342–354. 13867040

[B14] Berg AT, Mathern GW, Bronen RA, Fulbright RK, DiMario F, Testa FM, Levy SR (2009) Frequency, prognosis and surgical treatment of structural abnormalities seen with magnetic resonance imaging in childhood epilepsy. Brain 132:2785–2797. 10.1093/brain/awp187 19638447 PMC2759335

[B15] Berglind F, Ledri M, Sørensen AT, Nikitidou L, Melis M, Bielefeld P, Kirik D, Deisseroth K, Andersson M, Kokaia M (2014) Optogenetic inhibition of chemically induced hypersynchronized bursting in mice. Neurobiol Dis 65:133–141. 10.1016/j.nbd.2014.01.015 24491965

[B16] Bialer M, Johannessen SI, Koepp MJ, Levy RH, Perucca E, Perucca P, Tomson T, White HS (2020) Progress report on new antiepileptic drugs: a summary of the Fifteenth Eilat Conference on New Antiepileptic Drugs and Devices (EILAT XV). I. Drugs in preclinical and early clinical development. Epilepsia 61:2340–2364. 10.1111/epi.16725 33190243

[B17] Binding J, Ben Arous J, Léger J-F, Gigan S, Boccara C, Bourdieu L (2011) Brain refractive index measured in vivo with high-NA defocus-corrected full-field OCT and consequences for two-photon microscopy. Opt Express 19:4833–4847. 10.1364/OE.19.004833 21445119

[B18] Booker SA, Vida I (2018) Morphological diversity and connectivity of hippocampal interneurons. Cell Tissue Res 373:619–641. 10.1007/s00441-018-2882-2 30084021 PMC6132631

[B19] Botterill JJ, Lu YL, LaFrancois JJ, Bernstein HL, Alcantara-Gonzalez D, Jain S, Leary P, Scharfman HE (2019) An excitatory and epileptogenic effect of dentate gyrus mossy cells in a mouse model of epilepsy. Cell Rep 29:2875–2889.e6. 10.1016/j.celrep.2019.10.100 31775052 PMC6905501

[B20] Bouilleret V, Ridoux V, Depaulis A, Marescaux C, Nehlig A, Le Gal La Salle G (1999) Recurrent seizures and hippocampal sclerosis following intrahippocampal kainate injection in adult mice: electroencephalography, histopathology and synaptic reorganization similar to mesial temporal lobe epilepsy. Neuroscience 89:717–729. 10.1016/s0306-4522(98)00401-1 10199607

[B21] Bouilleret V, Loup F, Kiener T, Marescaux C, Fritschy JM (2000) Early loss of interneurons and delayed subunit-specific changes in GABA(A)-receptor expression in a mouse model of mesial temporal lobe epilepsy. Hippocampus 10:305–324. 10.1002/1098-1063(2000)10:3<305::AID-HIPO11>3.0.CO;2-I 10902900

[B22] Bui AD, Nguyen TM, Limouse C, Kim HK, Szabo GG, Felong S, Maroso M, Soltesz I (2018) Dentate gyrus mossy cells control spontaneous convulsive seizures and spatial memory. Science 359:787–790. 10.1126/science.aan4074 29449490 PMC6040648

[B23] Burette A, Zabel U, Weinberg RJ, Schmidt HH, Valtschanoff JG (2002) Synaptic localization of nitric oxide synthase and soluble guanylyl cyclase in the hippocampus. J Neurosci 22:8961–8970. 10.1523/JNEUROSCI.22-20-08961.2002 12388603 PMC6757692

[B24] Cela E, Sjöström PJ (2019) Novel optogenetic approaches in epilepsy research. Front Neurosci 13:947. 31551699 10.3389/fnins.2019.00947 PMC6743373

[B25] Chan D, Suk HJ, Jackson B, Milman NP, Stark D, Beach SD, Tsai LH (2021) Induction of specific brain oscillations may restore neural circuits and be used for the treatment of Alzheimer's disease. J Intern Med 290:993–1009. 10.1111/joim.13329 34156133

[B26] Chen R, Gore F, Nguyen QA, Ramakrishnan C, Patel S, Kim SH, Raffiee M, Kim YS, Hsueh B, Krook-Magnusson E, Soltesz I, Deisseroth K (2021) Deep brain optogenetics without intracranial surgery. Nat Biotechnol 39:161–164. 10.1038/s41587-020-0679-9 33020604 PMC7878426

[B27] Chen S, Weitemier AZ, Zeng X, He L, Wang X, Tao Y, Huang AJY, Hashimotodani Y, Kano M, Iwasaki H, Parajuli LK, Okabe S, Teh DBL, All AH, Tsutsui-Kimura I, Tanaka KF, Liu X, McHugh TJ (2018) Near-infrared deep brain stimulation via upconversion nanoparticle-mediated optogenetics. Science 359:679–684. 10.1126/science.aaq1144 29439241

[B28] Chiang CC, Ladas TP, Gonzalez-Reyes LE, Durand DM (2014) Seizure suppression by high frequency optogenetic stimulation using in vitro and in vivo animal models of epilepsy. Brain Stimul 7:890–899. 10.1016/j.brs.2014.07.034 25108607 PMC4259846

[B29] Christenson Wick Z, Krook-Magnuson E (2018) Specificity, versatility, and continual development: the power of optogenetics for epilepsy research. Front Cell Neurosci 12:151. 29962936 10.3389/fncel.2018.00151 PMC6010559

[B30] Christenson Wick Z, Krook-Magnuson E (2019) Seizing sequencing data to consider cell and circuit complexity. Epilepsy Curr 19:124–125. 10.1177/1535759719835658 30955433 PMC6610407

[B31] Christenson Wick Z, Leintz CH, Xamonthiene C, Huang BH, Krook-Magnuson E (2017) Axonal sprouting in commissurally projecting parvalbumin-expressing interneurons. J Neurosci Res 95:2336–2344. 10.1002/jnr.24011 28151564 PMC5540851

[B32] Christenson Wick Z, Tetzlaff MR, Krook-Magnuson E (2019) Novel long-range inhibitory nNOS-expressing hippocampal cells. Elife 8:e46816. 10.7554/eLife.46816 31609204 PMC6839902

[B33] Cohen J (1977) Statistical power analysis for the behavioral sciences, Ed 2. New York: Academic.

[B34] Cossart R, Bernard C, Ben-Ari Y (2005) Multiple facets of GABAergic neurons and synapses: multiple fates of GABA signalling in epilepsies. Trends Neurosci 28:108–115. 10.1016/j.tins.2004.11.011 15667934

[B35] DeFelipe J, et al. (2013) New insights into the classification and nomenclature of cortical GABAergic interneurons. Nat Rev Neurosci 14:202–216. 10.1038/nrn3444 23385869 PMC3619199

[B36] de Lanerolle NC, Brines ML, Kim JH, Williamson A, Philips MF, Spencer DD (1992) Neurochemical remodelling of the hippocampus in human temporal lobe epilepsy. Epilepsy Res Suppl 9:205–219. 1363041

[B37] de Tisi J, Bell GS, Peacock JL, McEvoy AW, Harkness WF, Sander JW, Duncan JS (2011) The long-term outcome of adult epilepsy surgery, patterns of seizure remission, and relapse: a cohort study. Lancet 378:1388–1395. 10.1016/S0140-6736(11)60890-8 22000136

[B38] Dimidschstein J, et al. (2016) A viral strategy for targeting and manipulating interneurons across vertebrate species. Nat Neurosci 19:1743–1749. 10.1038/nn.4430 27798629 PMC5348112

[B39] Duchowny M, Bhatia S (2014) Epilepsy: preserving memory in temporal lobectomy—are networks the key? Nat Rev Neurol 10:245–246. 10.1038/nrneurol.2014.67 24752125

[B40] Dudok B, Klein PM, Hwaun E, Lee BR, Yao Z, Fong O, Bowler JC, Terada S, Sparks FT, Szabo GG, Farrell JS, Berg J, Daigle TL, Tasic B, Dimidschstein J, Fishell G, Losonczy A, Zeng H, Soltesz I (2021) Alternating sources of perisomatic inhibition during behavior. Neuron 109:997–1012.e9. 10.1016/j.neuron.2021.01.003 33529646 PMC7979482

[B41] Dudok B, Klein PM, Soltesz I (2022) Toward understanding the diverse roles of perisomatic interneurons in epilepsy. Epilepsy Curr 22:54–60. 10.1177/15357597211053687 35233202 PMC8832350

[B42] Dzhala VI, Staley KJ (2003) Excitatory actions of endogenously released GABA contribute to initiation of ictal epileptiform activity in the developing hippocampus. J Neurosci 23:1840–1846. 10.1523/JNEUROSCI.23-05-01840.2003 12629188 PMC6741948

[B43] Ellender TJ, Raimondo JV, Irkle A, Lamsa KP, Akerman CJ (2014) Excitatory effects of parvalbumin-expressing interneurons maintain hippocampal epileptiform activity via synchronous afterdischarges. J Neurosci 34:15208–15222. 10.1523/JNEUROSCI.1747-14.2014 25392490 PMC4228130

[B44] England MJ, Liverman CT, Schultz AM, Strawbridge LM (2012) Epilepsy across the spectrum: promoting health and understanding. A summary of the Institute of Medicine report. Epilepsy Behav 25:266–276. 10.1016/j.yebeh.2012.06.016 23041175 PMC3548323

[B45] Farrell JS, Nguyen QA, Soltesz I (2019) Resolving the micro-macro disconnect to address core features of seizure networks. Neuron 101:1016–1028. 10.1016/j.neuron.2019.01.043 30897354 PMC6430140

[B46] Favero M, Sotuyo NP, Lopez E, Kearney JA, Goldberg EM (2018) A transient developmental window of fast-spiking interneuron dysfunction in a mouse model of dravet syndrome. J Neurosci 38:7912–7927. 10.1523/JNEUROSCI.0193-18.2018 30104343 PMC6125809

[B47] Fenno LE, Mattis J, Ramakrishnan C, Hyun M, Lee SY, He M, Tucciarone J, Selimbeyoglu A, Berndt A, Grosenick L, Zalocusky KA, Bernstein H, Swanson H, Perry C, Diester I, Boyce FM, Bass CE, Neve R, Huang ZJ, Deisseroth K (2014) Targeting cells with single vectors using multiple-feature Boolean logic. Nat Methods 11:763–772. 10.1038/nmeth.2996 24908100 PMC4085277

[B48] Francavilla R, Villette V, Luo X, Chamberland S, Muñoz-Pino E, Camiré O, Wagner K, Kis V, Somogyi P, Topolnik L (2018) Connectivity and network state-dependent recruitment of long-range VIP-GABAergic neurons in the mouse hippocampus. Nat Commun 9:5043. 10.1038/s41467-018-07162-5 30487571 PMC6261953

[B49] Freund TF, Buzsáki G (1996) Interneurons of the hippocampus. Hippocampus 6:347–470. 10.1002/(SICI)1098-1063(1996)6:4<347::AID-HIPO1>3.0.CO;2-I 8915675

[B50] Fuentealba P, Begum R, Capogna M, Jinno S, Márton LF, Csicsvari J, Thomson A, Somogyi P, Klausberger T (2008) Ivy cells: a population of nitric-oxide-producing, slow-spiking GABAergic neurons and their involvement in hippocampal network activity. Neuron 57:917–929. 10.1016/j.neuron.2008.01.034 18367092 PMC4487557

[B51] Greenfield LJ Jr (2013) Molecular mechanisms of antiseizure drug activity at GABAA receptors. Seizure 22:589–600. 10.1016/j.seizure.2013.04.015 23683707 PMC3766376

[B52] Gysbrechts B, Wang L, Trong NN, Cabral H, Navratilova Z, Battaglia F, Saeys W, Bartic C (2016) Light distribution and thermal effects in the rat brain under optogenetic stimulation. J Biophotonics 9:576–585. 10.1002/jbio.201500106 26192551

[B53] Harris KD, Hochgerner H, Skene NG, Magno L, Katona L, Bengtsson Gonzales C, Somogyi P, Kessaris N, Linnarsson S, Hjerling-Leffler J (2018) Classes and continua of hippocampal CA1 inhibitory neurons revealed by single-cell transcriptomics. PLoS Biol 16:e2006387. 10.1371/journal.pbio.2006387 29912866 PMC6029811

[B54] Hawkins J, Sarett LH (1957) On the efficacy of asparagine, glutamine, γ-aminobutyric acid and 2-pyrrolidinone in preventing chemically induced seizures in mice. Clin Chim Acta 2:481–484. 13500579 10.1016/0009-8981(57)90049-9

[B55] Holtkamp M (2018) Pharmacotherapy for refractory and super-refractory status epilepticus in adults. Drugs 78:307–326. 10.1007/s40265-017-0859-1 29368126

[B56] Hristova K, Martinez-Gonzalez C, Watson TC, Codadu NK, Hashemi K, Kind PC, Nolan MF, Gonzalez-Sulser A (2021) Medial septal GABAergic neurons reduce seizure duration upon optogenetic closed-loop stimulation. Brain 144:1576–1589. 10.1093/brain/awab042 33769452 PMC8219369

[B57] Iaccarino HF, Singer AC, Martorell AJ, Rudenko A, Gao F, Gillingham TZ, Mathys H, Seo J, Kritskiy O, Abdurrob F, Adaikkan C, Canter RG, Rueda R, Brown EN, Boyden ES, Tsai LH (2016) Gamma frequency entrainment attenuates amyloid load and modifies microglia. Nature 540:230–235. 10.1038/nature20587 27929004 PMC5656389

[B58] Jinno S, Klausberger T, Marton LF, Dalezios Y, Roberts JD, Fuentealba P, Bushong EA, Henze D, Buzsáki G, Somogyi P (2007) Neuronal diversity in GABAergic long-range projections from the hippocampus. J Neurosci 27:8790–8804. 10.1523/JNEUROSCI.1847-07.2007 17699661 PMC2270609

[B59] Jones M, McDermott B, Oliveira BL, O'Brien A, Coogan D, Lang M, Moriarty N, Dowd E, Quinlan L, Mc Ginley B, Dunne E, Newell D, Porter E, Elahi MA, M OH, Shahzad A (2019) Gamma band light stimulation in human case studies: groundwork for potential Alzheimer's disease treatment. J Alzheimers Dis 70:171–185. 10.3233/JAD-190299 31156180 PMC6700637

[B60] Kaneko K, Currin CB, Goff KM, Wengert ER, Somarowthu A, Vogels TP, Goldberg EM (2022) Developmentally regulated impairment of parvalbumin interneuron synaptic transmission in an experimental model of Dravet syndrome. Cell Rep 38:110580. 10.1016/j.celrep.2022.110580 35354025 PMC9003081

[B61] Klausberger T, Somogyi P (2008) Neuronal diversity and temporal dynamics: the unity of hippocampal circuit operations. Science 321:53–57. 10.1126/science.1149381 18599766 PMC4487503

[B62] Kotloski R, Gidal B (2022) Rescue treatments for seizure clusters. Neurol Clin 40:927–937. 10.1016/j.ncl.2022.03.016 36270699

[B63] Krnjević K (1983) GABA-mediated inhibitory mechanisms in relation to epileptic discharges. In: Basic mechanisms of neuronal hyperexcitability (Jasper H, van Gelder NM, eds), pp 249–280. New York: Alan R. Liss.

[B64] Krook-Magnuson E (2020) The devil's in the details: how to harness inhibition for seizure control. Epilepsy Curr 20:99–101. 10.1177/1535759720902078 32313505 PMC7160868

[B65] Krook-Magnuson E, Soltesz I (2015) Beyond the hammer and the scalpel: selective circuit control for the epilepsies. Nat Neurosci 18:331–338. 10.1038/nn.3943 25710834 PMC4340083

[B66] Krook-Magnuson E, Luu L, Lee SH, Varga C, Soltesz I (2011) Ivy and neurogliaform interneurons are a major target of μ-opioid receptor modulation. J Neurosci 31:14861–14870. 10.1523/JNEUROSCI.2269-11.2011 22016519 PMC3226788

[B67] Krook-Magnuson E, Armstrong C, Oijala M, Soltesz I (2013) On-demand optogenetic control of spontaneous seizures in temporal lobe epilepsy. Nat Commun 4:1376. 10.1038/ncomms2376 23340416 PMC3562457

[B68] Krook-Magnuson E, Ledri M, Soltesz I, Kokaia M (2014a) How might novel technologies such as optogenetics lead to better treatments in epilepsy? Adv Exp Med Biol 813:319–336. 25012388 10.1007/978-94-017-8914-1_26 PMC4968566

[B69] Krook-Magnuson E, Szabo GG, Armstrong C, Oijala M, Soltesz I (2014b) Cerebellar directed optogenetic intervention inhibits spontaneous hippocampal seizures in a mouse model of temporal lobe epilepsy. eNeuro 1:ENEURO.0005-14.2014. 10.1523/ENEURO.0005-14.2014 PMC4293636 25599088

[B70] Krook-Magnuson E, Armstrong C, Bui A, Lew S, Oijala M, Soltesz I (2015) *In vivo* evaluation of the dentate gate theory in epilepsy. J Physiol 593:2379–2388. 10.1113/JP270056 25752305 PMC4457198

[B71] Kuruba R, Hattiangady B, Parihar VK, Shuai B, Shetty AK (2011) Differential susceptibility of interneurons expressing neuropeptide Y or parvalbumin in the aged hippocampus to acute seizure activity. PLoS One 6:e24493. 10.1371/journal.pone.0024493 21915341 PMC3167860

[B72] Ladas TP, Chiang CC, Gonzalez-Reyes LE, Nowak T, Durand DM (2015) Seizure reduction through interneuron-mediated entrainment using low frequency optical stimulation. Exp Neurol 269:120–132. 10.1016/j.expneurol.2015.04.001 25863022 PMC4446206

[B73] Laxer KD, Trinka E, Hirsch LJ, Cendes F, Langfitt J, Delanty N, Resnick T, Benbadis SR (2014) The consequences of refractory epilepsy and its treatment. Epilepsy Behav 37:59–70. 10.1016/j.yebeh.2014.05.031 24980390

[B74] Lee SH, Marchionni I, Bezaire M, Varga C, Danielson N, Lovett-Barron M, Losonczy A, Soltesz I (2014) Parvalbumin-positive basket cells differentiate among hippocampal pyramidal cells. Neuron 82:1129–1144. 10.1016/j.neuron.2014.03.034 24836505 PMC4076442

[B75] Leshan RL, Greenwald-Yarnell M, Patterson CM, Gonzalez IE, Myers MG Jr (2012) Leptin action through hypothalamic nitric oxide synthase-1-expressing neurons controls energy balance. Nat Med 18:820–823. 10.1038/nm.2724 22522563 PMC3531967

[B76] Lévesque M, Chen LY, Etter G, Shiri Z, Wang S, Williams S, Avoli M (2019) Paradoxical effects of optogenetic stimulation in mesial temporal lobe epilepsy. Ann Neurol 86:714–728. 10.1002/ana.25572 31393618

[B77] Li T, Ren G, Lusardi T, Wilz A, Lan JQ, Iwasato T, Itohara S, Simon RP, Boison D (2008) Adenosine kinase is a target for the prediction and prevention of epileptogenesis in mice. J Clin Invest 118:571–582. 10.1172/JCI33737 18172552 PMC2157568

[B78] Löscher W, Schmidt D (2011) Modern antiepileptic drug development has failed to deliver: ways out of the current dilemma. Epilepsia 52:657–678. 10.1111/j.1528-1167.2011.03024.x 21426333

[B79] Lovett-Barron M, Losonczy A (2014) Behavioral consequences of GABAergic neuronal diversity. Curr Opin Neurobiol 26:27–33. 10.1016/j.conb.2013.11.002 24650501

[B80] Lu Y, Zhong C, Wang L, Wei P, He W, Huang K, Zhang Y, Zhan Y, Feng G, Wang L (2016) Optogenetic dissection of ictal propagation in the hippocampal-entorhinal cortex structures. Nat Commun 7:10962. 10.1038/ncomms10962 26997093 PMC4802168

[B81] Maglóczky Z, Freund TF (2005) Impaired and repaired inhibitory circuits in the epileptic human hippocampus. Trends Neurosci 28:334–340. 10.1016/j.tins.2005.04.002 15927690

[B82] Magloire V, Cornford J, Lieb A, Kullmann DM, Pavlov I (2019) KCC2 overexpression prevents the paradoxical seizure-promoting action of somatic inhibition. Nat Commun 10:1225. 10.1038/s41467-019-08933-4 30874549 PMC6420604

[B83] Maguire J (2018) Interneurons and the ictal orchestra. Epilepsy Curr 18:184–186. 10.5698/1535-7597.18.3.184 29950945 PMC6017675

[B84] Marx M, Haas CA, Haussler U (2013) Differential vulnerability of interneurons in the epileptic hippocampus. Front Cell Neurosci 7:167. 24098270 10.3389/fncel.2013.00167 PMC3787650

[B85] Miles R, Blaesse P, Huberfeld G, Wittner L, Kaila K (2012) Chloride homeostasis and GABA signaling in temporal lobe epilepsy. In: Jasper's basic mechanisms of the epilepsies, Ed 4 (Noebels JL, Avoli M, Rogawski MA, Olsen RW, Delgado-Escueta AV, eds). Bethesda, MD: National Center for Biotechnology Information. 22787654

[B86] Miyoshi G, Hjerling-Leffler J, Karayannis T, Sousa VH, Butt SJ, Battiste J, Johnson JE, Machold RP, Fishell G (2010) Genetic fate mapping reveals that the caudal ganglionic eminence produces a large and diverse population of superficial cortical interneurons. J Neurosci 30:1582–1594. 10.1523/JNEUROSCI.4515-09.2010 20130169 PMC2826846

[B87] Mouri G, Jimenez-Mateos E, Engel T, Dunleavy M, Hatazaki S, Paucard A, Matsushima S, Taki W, Henshall DC (2008) Unilateral hippocampal CA3-predominant damage and short latency epileptogenesis after intra-amygdala microinjection of kainic acid in mice. Brain Res 1213:140–151. 10.1016/j.brainres.2008.03.061 18455706

[B88] Moxon KA, Shahlaie K, Girgis F, Saez I, Kennedy J, Gurkoff GG (2019) From adagio to allegretto: the changing tempo of theta frequencies in epilepsy and its relation to interneuron function. Neurobiol Dis 129:169–181. 10.1016/j.nbd.2019.02.009 30798003

[B89] Muldoon SF, Villette V, Tressard T, Malvache A, Reichinnek S, Bartolomei F, Cossart R (2015) GABAergic inhibition shapes interictal dynamics in awake epileptic mice. Brain 138:2875–2890. 10.1093/brain/awv227 26280596

[B90] Muñoz-Manchado AB, Bengtsson Gonzales C, Zeisel A, Munguba H, Bekkouche B, Skene NG, Lönnerberg P, Ryge J, Harris KD, Linnarsson S, Hjerling-Leffler J (2018) Diversity of interneurons in the dorsal striatum revealed by single-cell RNA sequencing and PatchSeq. Cell Rep 24:2179–2190.e7. 10.1016/j.celrep.2018.07.053 30134177 PMC6117871

[B91] Nagaraj V, Lee ST, Krook-Magnuson E, Soltesz I, Benquet P, Irazoqui PP, Netoff TI (2015) Future of seizure prediction and intervention: closing the loop. J Clin Neurophysiol 32:194–206. 10.1097/WNP.0000000000000139 26035672 PMC4455045

[B92] Osawa S, Iwasaki M, Hosaka R, Matsuzaka Y, Tomita H, Ishizuka T, Sugano E, Okumura E, Yawo H, Nakasato N, Tominaga T, Mushiake H (2013) Optogenetically induced seizure and the longitudinal hippocampal network dynamics. PLoS One 8:e60928. 10.1371/journal.pone.0060928 23593349 PMC3622611

[B93] Paz JT, Davidson TJ, Frechette ES, Delord B, Parada I, Peng K, Deisseroth K, Huguenard JR (2013) Closed-loop optogenetic control of thalamus as a tool for interrupting seizures after cortical injury. Nat Neurosci 16:64–70. 10.1038/nn.3269 23143518 PMC3700812

[B94] Peixoto HM, Cruz RMS, Moulin TC, Leão RN (2020) Modeling the effect of temperature on membrane response of light stimulation in optogenetically-targeted neurons. Front Comput Neurosci 14:5. 10.3389/fncom.2020.00005 32116619 PMC7010719

[B95] Pelkey KA, Chittajallu R, Craig MT, Tricoire L, Wester JC, McBain CJ (2017) Hippocampal GABAergic Inhibitory Interneurons. Physiol Rev 97:1619–1747. 10.1152/physrev.00007.2017 28954853 PMC6151493

[B96] Peng Z, Zhang N, Wei W, Huang CS, Cetina Y, Otis TS, Houser CR (2013) A reorganized GABAergic circuit in a model of epilepsy: evidence from optogenetic labeling and stimulation of somatostatin interneurons. J Neurosci 33:14392–14405. 10.1523/JNEUROSCI.2045-13.2013 24005292 PMC3761049

[B97] Perucca P, Gilliam FG (2012) Adverse effects of antiepileptic drugs. Lancet Neurol 11:792–802. 10.1016/S1474-4422(12)70153-9 22832500

[B98] Riban V, Bouilleret V, Pham-Le BT, Fritschy JM, Marescaux C, Depaulis A (2002) Evolution of hippocampal epileptic activity during the development of hippocampal sclerosis in a mouse model of temporal lobe epilepsy. Neuroscience 112:101–111. 10.1016/S0306-4522(02)00064-7 12044475

[B99] Righes Marafiga J, Vendramin Pasquetti M, Calcagnotto ME (2021) GABAergic interneurons in epilepsy: more than a simple change in inhibition. Epilepsy Behav 121:106935. 10.1016/j.yebeh.2020.106935 32035792

[B100] Rungta RL, Osmanski BF, Boido D, Tanter M, Charpak S (2017) Light controls cerebral blood flow in naive animals. Nat Commun 8:14191. 10.1038/ncomms14191 28139643 PMC5290324

[B101] Sessolo M, Marcon I, Bovetti S, Losi G, Cammarota M, Ratto GM, Fellin T, Carmignoto G (2015) Parvalbumin-positive inhibitory interneurons oppose propagation but favor generation of focal epileptiform activity. J Neurosci 35:9544–9557. 10.1523/JNEUROSCI.5117-14.2015 26134638 PMC6605139

[B102] Sherman EM, Wiebe S, Fay-McClymont TB, Tellez-Zenteno J, Metcalfe A, Hernandez-Ronquillo L, Hader WJ, Jetté N (2011) Neuropsychological outcomes after epilepsy surgery: systematic review and pooled estimates. Epilepsia 52:857–869. 10.1111/j.1528-1167.2011.03022.x 21426331

[B103] Shin Y, Yoo M, Kim HS, Nam SK, Kim HI, Lee SK, Kim S, Kwon HS (2016) Characterization of fiber-optic light delivery and light-induced temperature changes in a rodent brain for precise optogenetic neuromodulation. Biomed Opt Express 7:4450–4471. 10.1364/BOE.7.004450 27895987 PMC5119587

[B104] Shiri Z, Manseau F, Lévesque M, Williams S, Avoli M (2016) Activation of specific neuronal networks leads to different seizure onset types. Ann Neurol 79:354–365. 10.1002/ana.24570 26605509 PMC4878884

[B105] Shiri Z, Lévesque M, Etter G, Manseau F, Williams S, Avoli M (2017) Optogenetic low-frequency stimulation of specific neuronal populations abates ictogenesis. J Neurosci 37:2999–3008. 10.1523/JNEUROSCI.2244-16.2017 28209738 PMC6596724

[B106] Shuman T, et al. (2020) Breakdown of spatial coding and interneuron synchronization in epileptic mice. Nat Neurosci 23:229–238. 10.1038/s41593-019-0559-0 31907437 PMC7259114

[B107] Soussi R, Boulland JL, Bassot E, Bras H, Coulon P, Chaudhry FA, Storm-Mathisen J, Ferhat L, Esclapez M (2014) Reorganization of supramammillary-hippocampal pathways in the rat pilocarpine model of temporal lobe epilepsy: evidence for axon terminal sprouting. Brain Struct Funct. 10.1007/s00429-014-0800-2 PMC4481331 24889162

[B108] Spuck S, Tronnier V, Orosz I, Schönweiler R, Sepehrnia A, Nowak G, Sperner J (2010) Operative and technical complications of vagus nerve stimulator implantation. Neurosurgery 67:489–494. 10.1227/NEU.0b013e3181f88867 21099577

[B109] Stieve BJ, Richner TJ, Krook-Magnuson C, Netoff TI, Krook-Magnuson E (2023) Optimization of closed-loop electrical stimulation enables robust cerebellar-directed seizure control. Brain 146:91–108. 10.1093/brain/awac051 35136942 PMC10202393

[B110] Streng ML, Krook-Magnuson E (2020) Excitation, but not inhibition, of the fastigial nucleus provides powerful control over temporal lobe seizures. J Physiol 598:171–187. 10.1113/JP278747 31682010 PMC6938547

[B111] Streng ML, Tetzlaff MR, Krook-Magnuson E (2021) Distinct fastigial output channels and their impact on temporal lobe seizures. J Neurosci 41:10091–10107. 10.1523/JNEUROSCI.0683-21.2021 34716233 PMC8660050

[B112] Sukhotinsky I, Chan AM, Ahmed OJ, Rao VR, Gradinaru V, Ramakrishnan C, Deisseroth K, Majewska AK, Cash SS (2013) Optogenetic delay of status epilepticus onset in an in vivo rodent epilepsy model. PLoS One 8:e62013. 10.1371/journal.pone.0062013 23637949 PMC3634849

[B113] Suzuki F, Junier MP, Guilhem D, Sørensen JC, Onteniente B (1995) Morphogenetic effect of kainate on adult hippocampal neurons associated with a prolonged expression of brain-derived neurotrophic factor. Neuroscience 64:665–674. 10.1016/0306-4522(94)00463-f 7715779

[B114] Szabo GG, Farrell JS, Dudok B, Hou WH, Ortiz AL, Varga C, Moolchand P, Gulsever CI, Gschwind T, Dimidschstein J, Capogna M, Soltesz I (2022) Ripple-selective GABAergic projection cells in the hippocampus. Neuron 110:1959–1977.e9. 10.1016/j.neuron.2022.04.002 35489331 PMC9233074

[B115] Tønnesen J, Kokaia M (2017) Epilepsy and optogenetics: can seizures be controlled by light? Clin Sci (Lond) 131:1605–1616. 10.1042/CS20160492 28667062

[B116] Trevelyan AJ, Schevon CA (2013) How inhibition influences seizure propagation. Neuropharmacology 69:45–54. 10.1016/j.neuropharm.2012.06.015 22722026

[B117] Tricoire L, Pelkey KA, Daw MI, Sousa VH, Miyoshi G, Jeffries B, Cauli B, Fishell G, McBain CJ (2010) Common origins of hippocampal Ivy and nitric oxide synthase expressing neurogliaform cells. J Neurosci 30:2165–2176. 10.1523/JNEUROSCI.5123-09.2010 20147544 PMC2825142

[B118] Tung JK, Shiu FH, Ding K, Gross RE (2018) Chemically activated luminopsins allow optogenetic inhibition of distributed nodes in an epileptic network for non-invasive and multi-site suppression of seizure activity. Neurobiol Dis 109:1–10. 10.1016/j.nbd.2017.09.007 28923596 PMC5696076

[B119] Twele F, Töllner K, Brandt C, Löscher W (2016) Significant effects of sex, strain, and anesthesia in the intrahippocampal kainate mouse model of mesial temporal lobe epilepsy. Epilepsy Behav 55:47–56. 10.1016/j.yebeh.2015.11.027 26736063

[B120] Varga C, Oijala M, Lish J, Szabo GG, Bezaire M, Marchionni I, Golshani P, Soltesz I (2014) Functional fission of parvalbumin interneuron classes during fast network events. Elife 3:e04006. 10.7554/eLife.04006 25375253 PMC4270094

[B121] Walker MC, Kullmann DM (2020) Optogenetic and chemogenetic therapies for epilepsy. Neuropharmacology 168:107751. 10.1016/j.neuropharm.2019.107751 31494141

[B122] Wamsley B, Fishell G (2017) Genetic and activity-dependent mechanisms underlying interneuron diversity. Nat Rev Neurosci 18:299–309. 10.1038/nrn.2017.30 28381833

[B123] Wang S, Kfoury C, Marion A, Lévesque M, Avoli M (2022) Modulation of in vitro epileptiform activity by optogenetic stimulation of parvalbumin-positive interneurons. J Neurophysiol 128:837–846. 10.1152/jn.00192.2022 36043700

[B124] Wang Y, Xu C, Xu Z, Ji C, Liang J, Wang Y, Chen B, Wu X, Gao F, Wang S, Guo Y, Li X, Luo J, Duan S, Chen Z (2017) Depolarized GABAergic signaling in subicular microcircuits mediates generalized seizure in temporal lobe epilepsy. Neuron 95:1221. 10.1016/j.neuron.2017.08.013 28858623

[B125] Weitz AJ, Fang Z, Lee HJ, Fisher RS, Smith WC, Choy M, Liu J, Lin P, Rosenberg M, Lee JH (2015) Optogenetic fMRI reveals distinct, frequency-dependent networks recruited by dorsal and intermediate hippocampus stimulations. Neuroimage 107:229–241. 10.1016/j.neuroimage.2014.10.039 25462689 PMC4409430

[B126] West PJ, Thomson K, Billingsley P, Pruess T, Rueda C, Saunders GW, Smith MD, Metcalf CS, Wilcox KS (2022) Spontaneous recurrent seizures in an intra-amygdala kainate microinjection model of temporal lobe epilepsy are differentially sensitive to antiseizure drugs. Exp Neurol 349:113954. 10.1016/j.expneurol.2021.113954 34922908 PMC8815304

[B127] Woodson W, Nitecka L, Ben-Ari Y (1989) Organization of the GABAergic system in the rat hippocampal formation: a quantitative immunocytochemical study. J Comp Neurol 280:254–271. 10.1002/cne.902800207 2925894

[B128] Yao Z, et al. (2021) A taxonomy of transcriptomic cell types across the isocortex and hippocampal formation. Cell 184:3222–3241.e26. 10.1016/j.cell.2021.04.021 34004146 PMC8195859

[B129] Zeidler Z, Brandt-Fontaine M, Leintz C, Krook-Magnuson C, Netoff T, Krook-Magnuson E (2018) Targeting the mouse ventral hippocampus in the intrahippocampal kainic acid model of temporal lobe epilepsy. eNeuro 5:ENEURO.0158-18.2018. 10.1523/ENEURO.0158-18.2018 PMC6102375 30131968

[B130] Zhang W, Yamawaki R, Wen X, Uhl J, Diaz J, Prince DA, Buckmaster PS (2009) Surviving hilar somatostatin interneurons enlarge, sprout axons, and form new synapses with granule cells in a mouse model of temporal lobe epilepsy. J Neurosci 29:14247–14256. 10.1523/JNEUROSCI.3842-09.2009 19906972 PMC2802278

[B131] Zumsteg D, Andrade DM, Wennberg RA (2006) Clinical electrophysiology factors indicative of intractability in patients with temporal lobe epilepsy. Adv Neurol 97:45–62. 16383114

